# DepPrior: integrating CRISPR dependency predictability with multi-omics reproducibility to prioritize candidate LUAD therapeutic targets

**DOI:** 10.3389/fphar.2026.1859926

**Published:** 2026-08-27

**Authors:** Feng Zhou, Cheng Wang, Lizhi Mo, Xuan Sun, Ji Zhang

**Affiliations:** Department of Thoracic Surgery, Changde First People’s Hospital, Changde, Hunan, China

**Keywords:** cohort reproducibility, CRISPR dependency, DepMap, DepScore, lung adenocarcinoma, tumor multi-omics

## Abstract

Lung adenocarcinoma (LUAD) remains molecularly heterogeneous, and many tumors lack clearly tractable vulnerabilities. We developed DepPrior, a computational framework that ranks candidate LUAD therapeutic targets by requiring concordant evidence of CRISPR dependency separability, molecular predictability, and cross-cohort expression/protein reproducibility. DepMap dependency scores were modeled from matched expression and copy-number features using linear and non-linear learners, and gene-level AUROC and R^2^ were combined into a heuristic DepScore. The final candidate set included FERMT2, CRKL, MYC, CHMP4B and related genes. The set formed a coherent tumor expression module in TCGA-LUAD, was strongly associated with proliferation-linked features, and showed rank-based concordance across GEO transcriptomic cohorts and CPTAC transcriptomic/proteomic resources. Five-fold cross-validation supported the ranking of non-linear models, although performance gains were moderate and should be interpreted as model-ranking evidence rather than as large effect-size proof. Orthogonal experiments in HCC827 cells showed modest but reproducible protein-level reductions after FERMT2 and CRKL knockdown, accompanied by a directionally stronger apoptosis-associated protein shift after combined suppression than after single perturbation. These findings support DepPrior as a reproducibility-oriented, hypothesis-generating approach for target nomination. Because cross-cohort expression concordance does not prove patient-tumor dependency conservation, and experimental validation was restricted to selected genes and cell-line systems without rescue or proliferation/clonogenic assays, the prioritized genes should be considered candidates for further perturbation, rescue, patient-derived model, and therapeutic tractability studies.

## 1 Introduction

Lung adenocarcinoma (LUAD) is the most common histological subtype of lung cancer and remains a major cause of cancer mortality worldwide (Travis, 2011; Zhang et al., 2023). Despite the clinical success of genotype-matched therapies in selected molecular subsets, a large proportion of LUAD tumors still lack clear therapeutic vulnerabilities, particularly beyond canonical driver-defined contexts (Ramalingam et al., 2020; Peters et al., 2017). Established genomic biomarkers, including single-gene mutations, copy-number events, and mutation burden summaries, do not necessarily capture the functional dependencies that sustain tumor fitness or shape context-specific treatment sensitivity (Tsher et al., 2017; Shi et al., 2024).

Large-scale CRISPR screening resources such as DepMap offer an important complementary view by directly quantifying gene dependency across diverse cancer cell lines (Tsher et al., 2017; Pacini et al., 2024). These datasets create an opportunity to move from descriptive tumor profiling to functionally informed target nomination (Akhoundova and Rubin, 2022; Garofalo et al., 2016). However, not all measured dependencies are equally useful for translational prioritization. Some dependencies are relatively stable and predictable from molecular context, whereas others are diffuse or strongly shaped by unmeasured variables. A practical challenge, therefore, is not only to identify strong dependency signals, but also to determine which candidate dependencies are sufficiently predictable, reproducible, and traceable to justify downstream biological interpretation (Kasif and Roberts, 2020).

A second unmet need concerns cross-context interpretation. Most dependency models are trained in cell lines, whereas the intended biological interpretation often concerns primary tumors (Shi et al., 2024; Yu et al., 2019). This creates an inherent transfer problem: molecular correlates of dependency *in vitro* may not imply functional dependency conservation in tumors (Yu et al., 2019; Lin and Sheltzer, 2020). In addition, associations observed in a single patient cohort may not replicate across expression platforms, proteomic measurements, or external datasets with different clinical composition (Raffield et al., 2020; Eldjarn et al., 2023). These issues motivate a more cautious, reproducibility-oriented framework for computational target prioritization, with explicit separation between target nomination and definitive dependency validation (Kasif and Roberts, 2020).

Several recent resources and prioritization pipelines already connect DepMap dependencies with tumor omics, drug sensitivity, or clinical annotation for target nomination (Shi et al., 2024; Pacini et al., 2024; Lin and Sheltzer, 2020). DepPrior differs from these approaches in a narrower and deliberately conservative way: it uses a conjunctive ranking rule that requires both dependency separability and molecular predictability before testing whether the resulting candidate set retains module-level structure across independent tumor datasets. Thus, the framework is intended to prioritize robust and reproducible candidates for follow-up, not to replace context-specific dependency discovery or to prove patient-tumor vulnerability from cell-line data alone. This distinction is important because the final candidate set is strongly proliferation-linked and should be interpreted as a structured, reproducible candidate module rather than as a wholly new LUAD vulnerability class.

Here, we applied DepPrior to LUAD by integrating DepMap CRISPR dependency data with TCGA-LUAD multi-omics and external GEO/CPTAC cohorts. We benchmarked representative machine-learning models for gene-wise dependency prediction, quantified dependency separability and predictability, and combined these features into a pragmatic prioritization index termed DepScore. We then derived a compact candidate LUAD target set, evaluated its expression coherence, clinical associations, lineage-related patterns, and cross-platform reproducibility, and performed orthogonal perturbation-based experiments for selected representative genes. Throughout the manuscript, we interpret the output as a hypothesis-generating candidate set rather than as definitive evidence of druggable or patient-conserved dependencies.

## 2 Methods

### 2.1 Study design

We implemented a multi-step framework to prioritize a candidate LUAD therapeutic target set and evaluate its reproducibility across cohorts, omics layers, and selected experimental models. To reduce circular interpretation, the workflow was separated into four phases. The discovery/modeling phase used DepMap dependency and matched molecular feature matrices only. The prioritization phase ranked genes using pre-specified AUROC, R^2^, and DepScore criteria. The tumor-level and external reproducibility phase used TCGA, GEO, and CPTAC data after the candidate set had been fixed. The experimental-support phase tested selected representative genes in LUAD cell models. External tumor cohorts and immunoblotting results were not used to train the prediction models or to calculate DepScore.

The full workflow consisted of six operational stages: (i) assembly and harmonization of DepMap dependency and molecular feature matrices; (ii) assembly of TCGA-LUAD tumor multi-omics and clinical data; (iii) gene-wise dependency prediction benchmarking across representative machine-learning models; (iv) prioritization of candidate genes using a composite DepScore derived from dependency separability and predictability; (v) tumor-level and cross-cohort reproducibility assessment using TCGA, GEO, and CPTAC data; and (vi) orthogonal perturbation-based testing of selected candidate genes in LUAD cell models. The analysis emphasized reproducibility and coherence of the resulting candidate target set while treating the experimental results as initial support for selected genes rather than definitive proof of broad dependency conservation in patient tumors.

### 2.2 Data sources

#### 2.2.1 TCGA-LUAD cohort

TCGA-LUAD gene expression, clinical annotation mutation calls, copy-number profiles, and DNA methylation data were obtained from publicly accessible TCGA/GDC resources (Giordano, 2014; Zhang et al., 2021; Mounir et al., 2019). RNA-seq data were converted to log2 (TPM+1) for downstream analyses. Somatic mutation profiles were summarized from MAF files. Gene-level copy-number values were obtained by mapping segment-level values to gene loci. Promoter methylation was summarized from HM450 probes annotated to promoter-associated regions. When multiple modalities were available for a given patient, samples were aligned by TCGA barcode and primary tumor samples were prioritized. Overall survival (OS) time and event status were derived from death and last follow-up annotations.

#### 2.2.2 DepMap cohort

DepMap gene dependency matrices and matched molecular feature matrices were obtained from public DepMap releases (Ghandi et al., 2019; Pacini et al., 2021). For prediction modeling, gene expression and copy-number profiles were used as input features. LUAD-restricted analyses used cell lines annotated as lung adenocarcinoma, whereas broader screening and benchmarking analyses used all available cancer cell lines with matched dependency and molecular data.

#### 2.2.3 GEO and CPTAC external reproducibility datasets

Independent LUAD transcriptomic cohorts were collected from GEO, including GSE50081 (Der et al., 2014) and GSE72094 (Schabath et al., 2016). These datasets were selected because they include lung adenocarcinoma samples, public sample-level expression matrices, sufficient coverage of the candidate genes, and independent processing histories relative to TCGA. CPTAC-derived transcriptomic and proteomic matrices were included because they provide an orthogonal proteogenomic layer for assessing whether the expression-defined candidate set is preserved at the RNA and protein levels (Gillette et al., 2020; Li et al., 2023). Because absolute scaling differs substantially across cohorts and platforms, downstream external analyses focused primarily on rank-based concordance and cohort-specific signature construction rather than direct comparison of raw expression values.

### 2.3 Feature preprocessing and harmonization

Expression and copy-number matrices from DepMap were concatenated into a unified feature matrix after removal of non-numeric and all-missing columns. Features were standardized to zero mean and unit variance. To reduce dimensionality and multicollinearity, principal component analysis (PCA) was applied, and the top 256 principal components were used for model fitting in the benchmark workflow. Across datasets, gene identifiers were harmonized to HGNC gene symbols wherever possible. External comparisons used only the intersection of genes available in the paired cohorts being compared. This implementation detail was confirmed from the provided analysis notebooks.

### 2.4 Gene-wise dependency prediction

#### 2.4.1 Problem formulation

For each candidate gene g, we modeled its dependency score y_g across cell lines as a function of matched molecular features X. This was treated primarily as a regression problem, with gene-wise R^2^ and RMSE used to quantify continuous predictability. To capture whether a gene also separated strongly dependent from non-dependent cell lines, dependency scores were additionally binarized and AUROC was computed for each gene. The two metrics were used because they answer different questions: R^2^/RMSE evaluate how well the magnitude of dependency can be predicted, whereas AUROC evaluates whether the model can distinguish a high-dependency subgroup. Genes supported by only one of these two views were interpreted more cautiously.

#### 2.4.2 Definition of dependent state for AUROC

For AUROC calculation, dependency scores were binarized using a pre-specified threshold of gene effect < −0.5, which was treated as the dependent/essential state in the current implementation. This threshold was fixed before score integration and used consistently across candidate genes.

#### 2.4.3 Data splitting and model comparison

For model benchmarking, all candidate models were evaluated using identical train/test partitions for a given gene to ensure fair comparison. In the notebook implementation, the main held-out benchmark used an 80/20 split with random_state = 42; LUAD-specific downstream tasks used a saved train/validation/test split generated from the same seed. Preprocessing steps, including feature filtering, scaling, and PCA, were fitted on training data only and then applied to held-out data. We compared representative linear and non-linear models implemented mainly in scikit-learn (Pedregosa et al., 2011), including Ridge, Lasso, Elastic Net, Random Forest (Breiman, 2001), ExtraTrees (Geurts et al., 2006), Gradient Boosting (Friedman, 2001), SVR with radial basis kernel (Cortes and Vapnik, 1995), KNN, and XGBoost where available (Chen and Guestrin, 2016). The current study did not perform exhaustive nested hyperparameter optimization; instead, fixed or minimally tuned settings were used to estimate relative gene-wise predictability under a reproducible benchmark.

#### 2.4.4 Statistical comparison of models

Model performance was summarized by mean AUROC, mean R^2^, and RMSE across the benchmarked genes. For gene-wise paired comparisons between selected models, paired t-tests were used on per-gene performance differences. These comparisons were used descriptively to assess systematic model improvement, not to claim universal optimality of any one algorithm. Confidence intervals and fold-level variability were reported where available, and model-ranking claims were interpreted together with five-fold cross-validation results rather than a single split alone.

#### 2.4.5 Five-fold cross-validation for each model

To evaluate the robustness of model ranking beyond a single held-out split, each benchmarked model was additionally assessed using five-fold cross-validation on the high-signal gene benchmark set. For each gene, cell lines with matched dependency and molecular feature data were partitioned into five folds using shuffled K-fold splitting with a fixed random seed. Within each fold, preprocessing steps, including feature filtering, z-score scaling, and PCA when used, were fitted on the training portion only and then applied to the corresponding validation fold to prevent information leakage.

Regression performance was quantified using R^2^ and RMSE, and classification-style predictability was quantified using AUROC after binarizing dependency at gene effect < −0.5. For each model, fold-specific metrics were first averaged across benchmarked genes and then summarized across the five folds as mean ± standard deviation. This analysis was used as a robustness assessment of model ranking rather than as a separate model-selection stage.

### 2.5 Definition of high-signal genes and DepScore

#### 2.5.1 High-signal candidate pool

We first defined a high-signal candidate pool by requiring both acceptable separability and predictability. In the current analysis, genes with AUROC ≥0.80 and R^2^ ≥ 0.10 were considered high-signal candidates. These cutoffs were chosen as conservative operating thresholds to require both strong binary separability and non-trivial continuous predictability; they are not claimed to be biologically optimal universal cutoffs. Threshold sensitivity was assessed by scanning AUROC and R^2^ combinations in Supplementary Figure S1. Because the present manuscript is based on the analyzable gene set available in the provided implementation, these candidates should be interpreted as a screened candidate pool rather than a definitive genome-wide catalog.

#### 2.5.2 DepScore

To jointly reward dependency separability and predictability, we defined a pragmatic prioritization index (Equation 1):

$$\text{DepScore}\_g=\text{AUROC}\_g\times \max\left(R^{2}\_g,0\right)$$ (1)

This score was designed as a heuristic prioritization metric rather than a formal statistical estimator. It does not represent a probability, likelihood, or calibrated effect size. The multiplicative form was selected because it acts as a conjunctive rule: a gene receives a high score only when both AUROC and positive R^2^ are favorable, whereas a weighted sum can rank a gene highly if only one component is strong. To address sensitivity to this choice, alternative equal-weight, rank-based, and harmonic-mean scoring schemes were evaluated as robustness checks and used to identify scoring-sensitive genes. DepScore was retained as the primary rule because it is transparent, penalizes one-dimensional signals, and produced a compact candidate set enriched for recurrent high-priority genes across sensitivity analyses. Genes in the high-signal pool were ranked by DepScore, and the final candidate LUAD therapeutic target set was defined as the top 12 genes.

### 2.6 Construction of the cohort-specific DepSignature

Given a selected gene set S, we constructed a cohort-specific DepSignature using standardized gene expression values. For each gene g in S, expression was z-scored within the cohort (Equation 2):

$$z\_i,g=\left(x\_i,g-\mu \_g\right)\;/\;\sigma \_g$$ (2)

where x_i,g is the expression of gene g in sample i, and μ_g and σ_g denote the within-cohort mean and standard deviation of gene g. The sample-level signature was then defined as follows (Equation 3):

$$\text{DepSignature}\_i=\left|S\right|{\;}^{-1}\times \Sigma \_g\in S\;z\_i,g$$ (3)

This procedure was performed independently in each cohort to avoid cross-cohort scaling artifacts.

### 2.7 TCGA tumor-level analyses

#### 2.7.1 Survival analysis

Overall survival was analyzed using Kaplan–Meier curves and Cox proportional hazards models. For visualization, tumors were stratified by median or tertile splits of the cohort-specific signature. For regression analysis, the signature was treated as a continuous covariate. Multivariable models included age and stage-related covariates when available. Given the retrospective nature of the analysis, survival associations were interpreted as exploratory and hypothesis-generating.

#### 2.7.2 Association with clinicobiological variables

We examined associations between the DepSignature and proliferation markers (including MKI67 and TOP2A), pathologic T/N stage, and simple immune-related marker composites. Spearman correlation was used for continuous variables, Mann–Whitney U test for two-group comparisons, and Kruskal–Wallis test for multi-group comparisons.

#### 2.7.3 Mutation-specific comparisons

For canonical LUAD driver genes, tumors were grouped into mutant and wild-type categories. Mutation-specific signature differences were tested using Mann–Whitney U tests. Genes with fewer than 10 mutant tumors were considered low-frequency events and interpreted descriptively rather than inferentially. Because mutation-specific and clinicobiological association analyses were exploratory, nominal p values are reported and interpreted descriptively rather than as confirmatory evidence.

#### 2.7.4 Statistical reporting, multiplicity, and survival diagnostics

All statistical tests were two-sided unless otherwise specified. Survival analyses used Kaplan-Meier curves with log-rank tests for visualization and Cox proportional hazards models for regression. Cox models report hazard ratios with 95% confidence intervals where model coefficients are presented, and proportional-hazards assumptions were assessed using Schoenfeld-residual diagnostics implemented in the survival-analysis workflow (Davidson-Pilon, 2019). Mutation subgroup, clinicobiological, and correlation analyses were treated as exploratory because they were not used to select the candidate target set. Accordingly, nominal p values are shown in the main figures for readability, while families of related exploratory tests were interpreted descriptively and, where tabulated, accompanied by Benjamini-Hochberg false-discovery-rate estimates (Benjamini and Hochberg, 1995).

### 2.8 Cross-cohort and cross-omics reproducibility assessment

External assessment focused on module reproducibility rather than on direct proof of tumor dependency. For each cohort, we computed mean expression ranks of selected genes and quantified concordance with TCGA using Spearman correlation. Similar rank-based analyses were used for CPTAC transcriptomic and proteomic datasets. Because survival endpoints vary across retrospective cohorts, external assessment emphasized reproducibility of the candidate target set across measurement platforms and data sources.

### 2.9 Robustness analyses

We evaluated robustness of the high-signal pool by scanning combinations of AUROC and R^2^ thresholds and computing overlap of selected genes across threshold settings. We also compared the primary product-based DepScore with equal-weight scaled sums, rank aggregation, and harmonic-mean style composite scores to determine which genes were stable across ranking rules and which were scoring-rule sensitive.

To determine whether the observed candidate target set performed better than expected by chance, we generated matched-size random gene sets and compared their mean AUROC, mean R^2^, mean DepScore, and TCGA-related association statistics against those of the observed candidate target set. These analyses were used to assess score stability and guard against arbitrary threshold-driven selection.

### 2.10 Software and reproducibility

Analyses were implemented in Python 3.12.6 and R 4.5.2 using standard scientific computing and survival-analysis libraries, including pandas, NumPy, scikit-learn, XGBoost, lifelines, survival, and survminer where applicable. Data preprocessing, feature alignment, candidate gene ranking, cohort-level signature analysis, model benchmarking, and figure generation were organized as a reproducible mixed Python/R workflow. Random seeds were fixed for major stochastic steps, including NumPy initialization (seed = 0), PCA and tree-based models where applicable (random_state = 0), train/test splitting (random_state = 42), and five-fold cross-validation. Code notebooks are deposited at https://github.com/jizhang0114/DepPrior1, with repository documentation organized to include a top-level README, run-order instructions, a dataset dictionary, environment specifications, package-version/sessionInfo collection scripts, random-seed documentation, run-log guidance, and a workflow diagram after removal of local absolute paths and restricted raw-data files.

### 2.11 Orthogonal perturbation-based support in LUAD cell lines

To provide orthogonal support for the computationally prioritized candidate target set, we selected representative LUAD cell lines with relatively high or low DepSignature values for perturbation experiments. HCC827, H1975, and PC9 were used as signature-high models, whereas A549 and H1437 were used as signature-low models; BEAS-2B cells served as a non-malignant bronchial epithelial reference. FERMT2 and CRKL were chosen because they ranked highly and represented distinct biological axes within the prioritized module. These experiments were designed as feasibility and molecular-support experiments for selected genes, not as validation of the entire top-ranked set.

Cells were transfected with pooled control siRNA, siFERMT2, siCRKL, or a dual-knockdown combination using a lipid-based transfection reagent according to the manufacturer’s protocol. Knockdown efficiency was evaluated 48 h after transfection by qRT-PCR across the experimental panel and by immunoblotting in HCC827 cells. qRT-PCR was performed in triplicate for each condition. The qRT-PCR summary values and replicate-level raw Ct/calculation tables are provided in Supplementary Tables S5, S6. The complete instrument-generated qRT-PCR source-data package, including SDS7500 raw export files, run metadata, log files, plate setup information, raw Ct tables, amplification and melt-curve outputs, QC summaries, and replicate-level DeltaCt/DeltaDeltaCt/RQ calculation files, has been deposited in the public GitHub repository at https://github.com/jizhang0114/DepPrior1 as qPCR_raw_data.zip.

For Western blot analysis, total protein was extracted for baseline protein assessment from HCC827, A549, and BEAS-2B cells, and for post-transfection analysis from HCC827 cells transfected with siNC, siFERMT2, siCRKL, or combined siFERMT2 + siCRKL. Equal amounts of protein were separated by SDS-PAGE and transferred to PVDF membranes. Membranes were blocked and incubated with primary antibodies against FERMT2, CRKL, cleaved PARP, cleaved caspase-3, BCL-2, BAX, and beta-actin, followed by HRP-conjugated secondary antibodies. The baseline-expression blots assessed FERMT2 and CRKL in HCC827, A549, and BEAS-2B cells; the knockdown-confirmation blots assessed FERMT2 and CRKL across the four HCC827 transfection groups; and the apoptosis-response blots assessed cleaved PARP, cleaved caspase-3, BCL-2, and BAX across the same four HCC827 transfection groups.

PVDF membranes were incubated with enhanced chemiluminescence substrate and imaged on a chemiluminescence documentation system in grayscale acquisition mode. The detector records chemiluminescent photon-emission intensity rather than the physical color of the membrane. During acquisition or export, the software can display the same signal either as light bands on a dark background or as an inverted grayscale image with dark bands on a white background. Accordingly, the white-background raw/source files included in the supplementary data reflect the display polarity or lookup-table setting used for export of the chemiluminescent image, not colorimetric development or a different membrane. No bands were selectively altered before densitometry. Band intensities were quantified from three independent biological replicates; target-protein band area was normalized to the corresponding beta-actin loading-control band from the same lane. Baseline FERMT2 and CRKL protein levels were expressed relative to BEAS-2B, whereas knockdown-efficiency and apoptosis-marker values were expressed relative to the siNC control group. Quantified data are presented as mean±SD and were analyzed using one-way ANOVA followed by Dunnett’s multiple-comparisons test where appropriate. Uncropped membrane/source images, processed blot files, and densitometry graph exports/calculation summaries from the triplicate experiments were reorganized into consolidated supplementary source-data plates for review.

## 3 Results

### 3.1 Study framework and integrated LUAD resources

**FIGURE 1:**
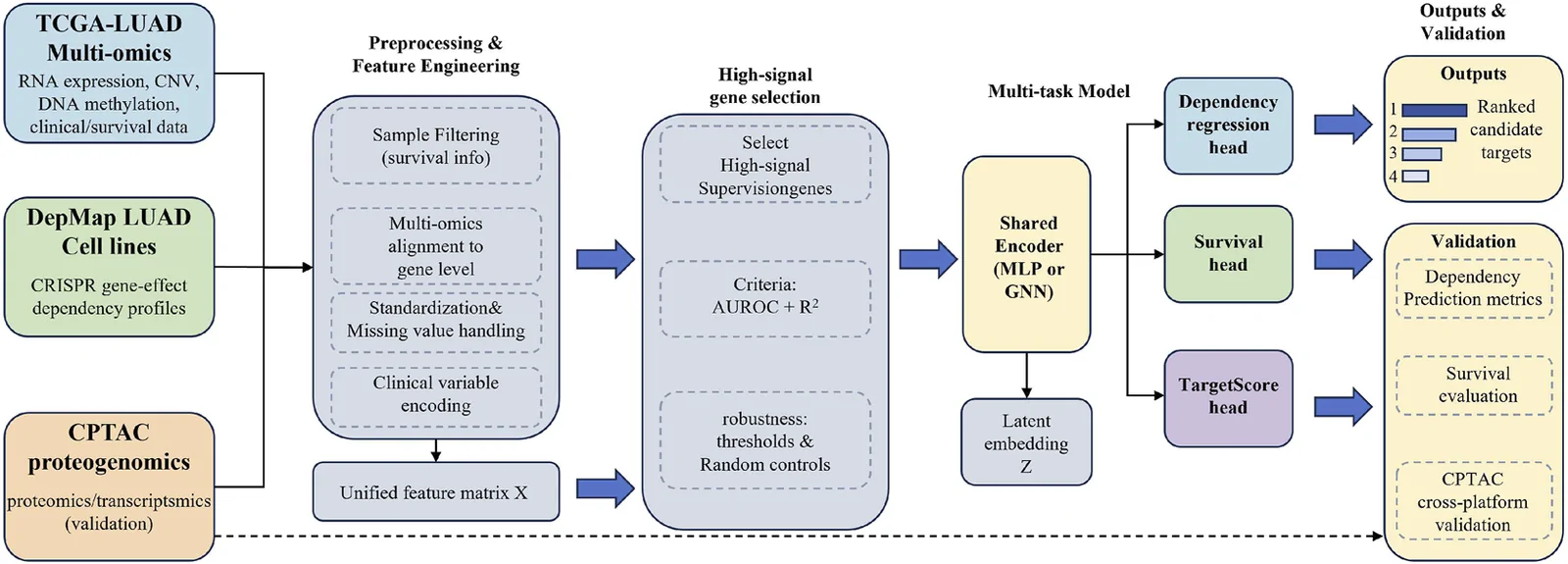
Overview of the predictability-guided framework for LUAD candidate therapeutic target prioritization. The single flowchart integrates TCGA-LUAD tumor multi-omics, DepMap LUAD CRISPR gene-effect data, and CPTAC proteogenomic data; summarizes preprocessing and feature engineering, high-signal gene selection, and multi-task modeling; and outputs ranked candidate targets with dependency, survival, and cross-platform validation.

We developed an integrative framework that links LUAD tumor multi-omics (TCGA-LUAD), cell-line genetic dependencies (DepMap), proteogenomic resources (CPTAC), and orthogonal perturbation-based experiments in selected LUAD models to define and assess a dependency-derived LUAD candidate target set. The computational workflow includes multi-omics preprocessing, feature engineering, high-signal gene selection based on dependency predictability, model benchmarking, definition of DepScore and downstream derivation of a candidate target set, and multi-layer reproducibility assessment across cohorts and omics layers (Figure 1). Orthogonal perturbation-based experiments were then performed as a downstream support step for selected targets.

**FIGURE 2:**
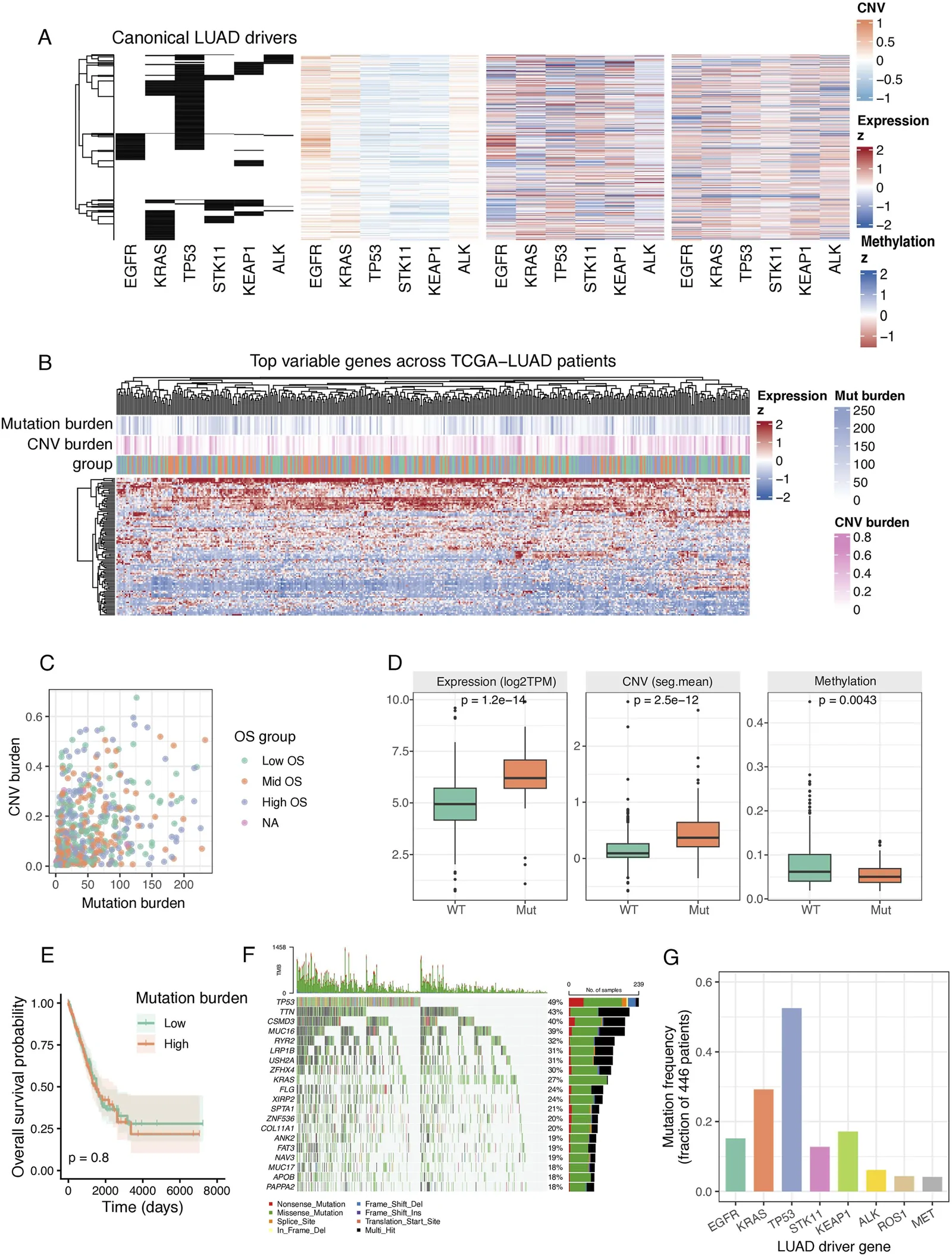
Molecular heterogeneity and canonical driver features in TCGA-LUAD. **(A)** Distribution of tumor mutation burden across TCGA-LUAD samples. **(B)** Genome-wide copy-number variation patterns. **(C)** Global DNA methylation heterogeneity across tumors. **(D)** Coordinated changes in expression, copy number and methylation in EGFR-mutant tumors. **(E)** Kaplan–Meier overall survival stratified by mutation burden. **(F)** Landscape of somatic mutations in TCGA-LUAD. **(G)** Frequencies of recurrent LUAD driver mutations.

Using TCGA-LUAD, we first confirmed canonical LUAD driver patterns across mutation/CNV/expression/methylation and summarized inter-patient heterogeneity (Figures 2A–C). As a representative example, EGFR-mutant tumors showed increased EGFR expression (p = 1.2 × 10^−14^) and elevated CNV signal (p = 2.5 × 10^−12^), with a corresponding methylation shift (p = 0.0043) (Figure 2D). In contrast, stratifying patients by global mutation burden did not yield clear survival separation (log-rank p = 0.8) (Figure 2E), motivating a multi-gene candidate set approach rather than burden-based prognostication. We further summarized the mutational landscape and canonical driver frequencies (Figures 2F,G).

### 3.2 Benchmarking dependency prediction models establishes a robust baseline for candidate target prioritization

**FIGURE 3:**
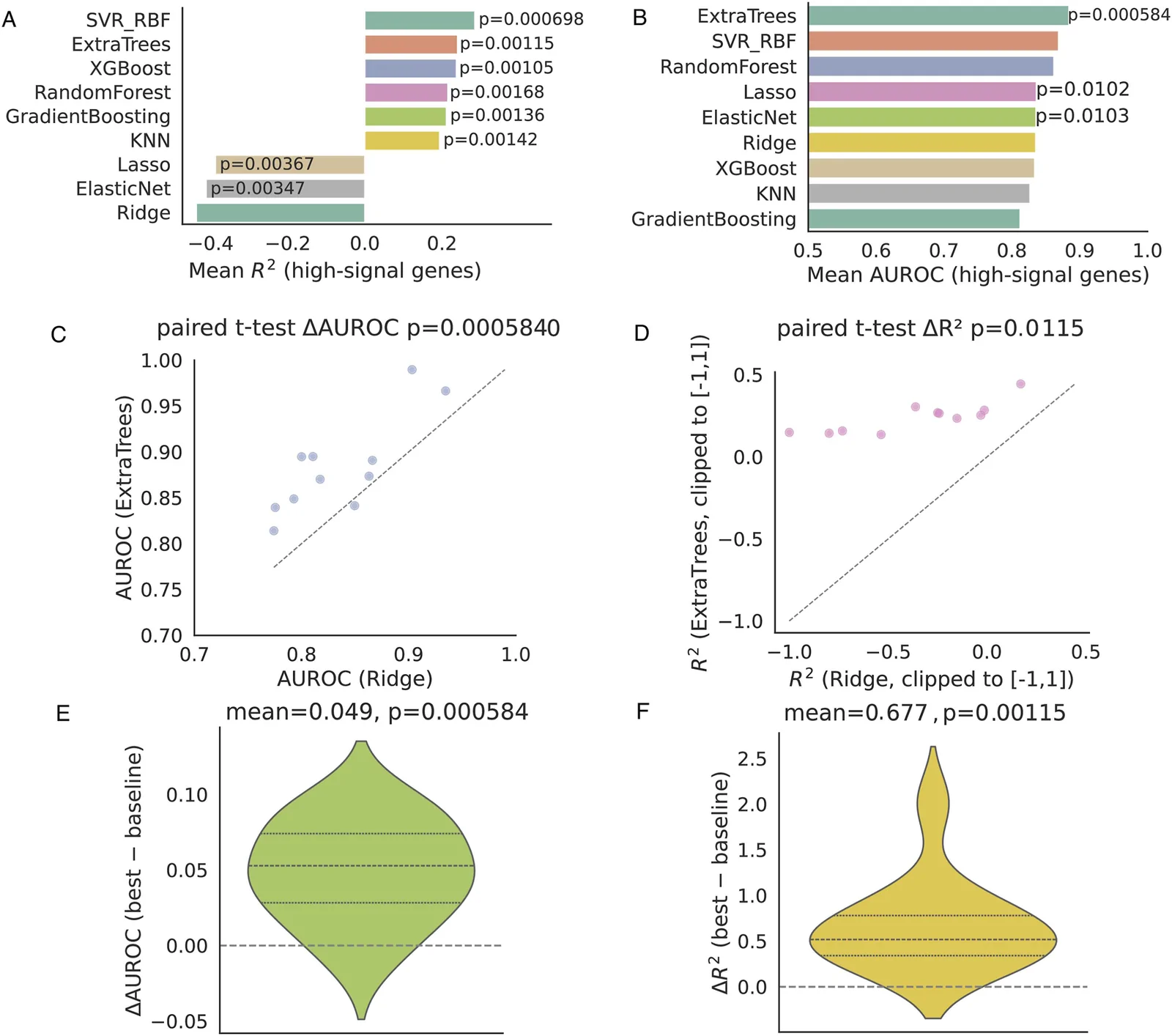
Benchmarking machine-learning models for gene dependency prediction in DepMap. **(A)** Mean AUROC of benchmarked models evaluated on high-signal genes. **(B)** Mean R^2^ values across models on held-out test sets. **(C)** Gene-wise AUROC comparison between ExtraTrees and Ridge regression. **(D)** Gene-wise R^2^ comparison between ExtraTrees and Ridge regression. **(E)** Distribution of AUROC improvements across genes. **(F)** Distribution of R^2^ improvements across genes.

To ensure that downstream target prioritization rests on reliable predictive performance, we benchmarked multiple machine-learning models on a high-signal gene subset in DepMap (Figure 3). Non-linear models consistently outperformed linear baselines, with ExtraTrees and SVR_RBF achieving the best overall performance on high-signal genes (mean R^2^ and mean AUROC; Figures 3A,B). Gene-wise paired comparisons indicated improved AUROC and R^2^ for ExtraTrees relative to Ridge (Figures 3C,D). Because held-out gene-wise contrasts and five-fold cross-validation summarize different estimands, the improvement distributions are interpreted descriptively as modest but systematic model-ranking evidence rather than as a single universal ΔR^2^ effect size (Figures 3E,F).

**FIGURE 4:**
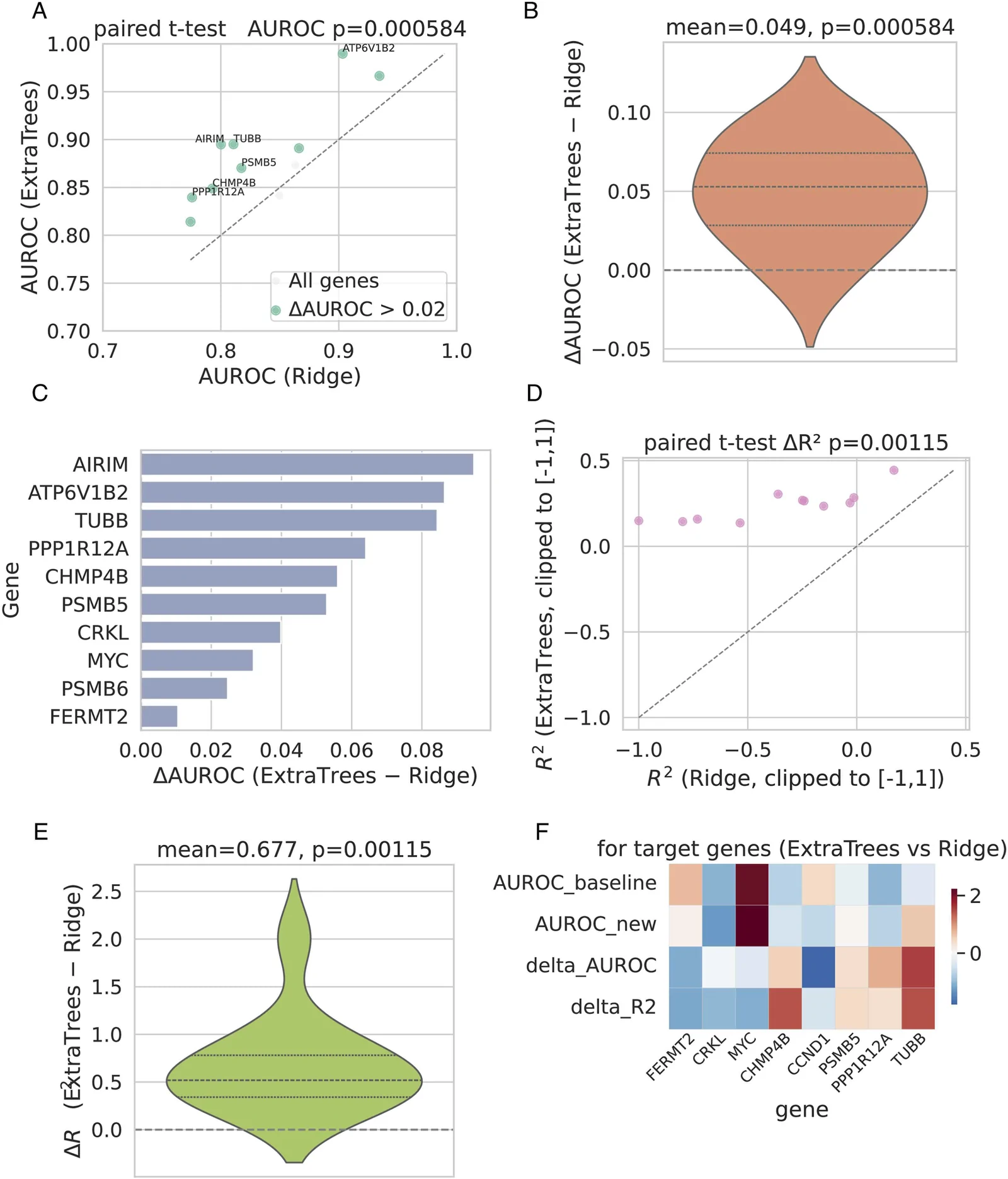
Non-linear models improve dependency predictability for DepPrior-prioritized candidate target genes. **(A)** Gene-wise comparison of AUROC values between Ridge and ExtraTrees models, shown as a descriptive paired comparison. **(B)** Distribution of AUROC improvements (ΔAUROC) from Ridge to ExtraTrees across candidate target genes. **(C)** Ranking of candidate target genes by ΔAUROC (ExtraTrees − Ridge). **(D)** Gene-wise comparison of R^2^ values between Ridge and ExtraTrees models after clipping to [−1, 1], shown as a descriptive paired comparison. **(E)** Distribution of *R* ^2^ improvements (ΔR^2^) from Ridge to ExtraTrees across candidate target genes. **(F)** Heatmap summarizing baseline and improved predictability metrics (AUROC and R^2^) for candidate target genes. **(A)** Gene-wise comparison of AUROC values between Ridge and ExtraTrees models, with a paired t-test indicated. **(B)** Distribution of AUROC improvements (ΔAUROC) from Ridge to ExtraTrees across candidate target genes. **(C)** Ranking of candidate target genes by ΔAUROC (ExtraTrees − Ridge). **(D)** Gene-wise comparison of R^2^ values between Ridge and ExtraTrees models after clipping to [−1, 1]. **(E)** Distribution of R^2^ improvements (ΔR^2^) from Ridge to ExtraTrees across candidate target genes. **(F)** Heatmap summarizing baseline and improved predictability metrics (AUROC and R^2^) for candidate target genes.

Focusing on the selected candidate target set, the same direction of improvement was observed but was treated as a subset-specific descriptive analysis. ExtraTrees tended to increase AUROC relative to Ridge, with the largest gains concentrated among candidate target genes (Figures 4A–C), and R^2^ changes showed the same directionality after clipping extreme values (Figures 4D,E). Candidate-set p values and effect sizes were not assumed to be identical to those from the full high-signal benchmark; they should be read as separate paired summaries for the smaller prioritized subset. The consolidated heatmap in Figure 4F summarizes baseline AUROC, improved AUROC, ΔAUROC, and ΔR^2^ across candidate target genes, providing a methodologically grounded baseline for dependency prediction.

### 3.3 Pan-DepMap landscape defines a compact LUAD candidate target set via DepScore

**FIGURE 5:**
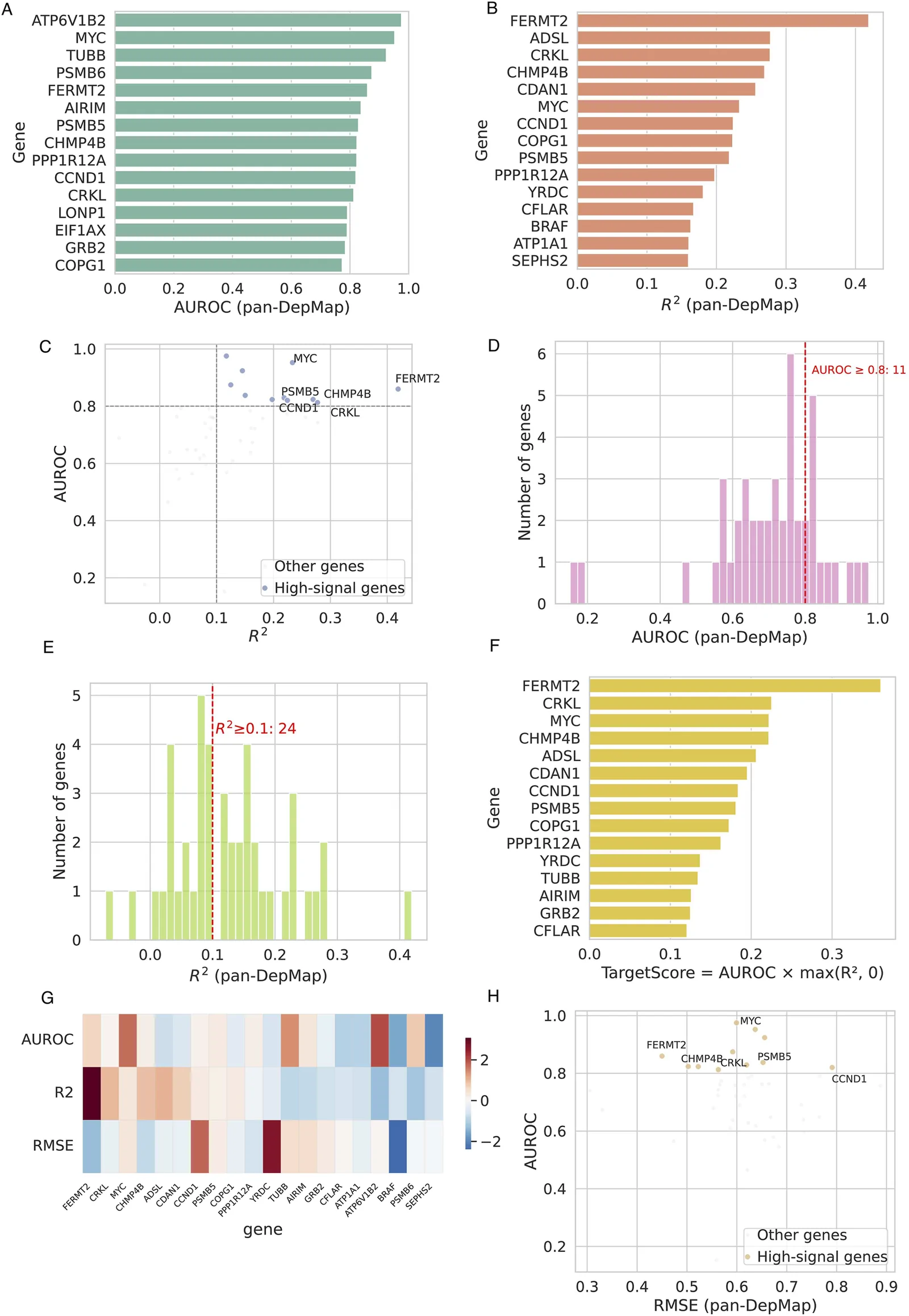
Pan-DepMap dependency predictability landscape and DepScore-based gene prioritization. **(A)** Top-ranked genes by AUROC across pan-DepMap. **(B)** Top-ranked genes by R^2^ across pan-DepMap. **(C)** AUROC–R^2^ scatterplot highlighting high-performance genes. **(D)** Thresholded view of genes with strong separability and predictability. **(E)** Overlap of high-AUROC and high-R^2^ gene sets. **(F)** DepScore-ranked genes defining the LUAD candidate target set. **(G)** Heatmap summarizing AUROC, R^2^ and RMSE for candidate target genes. **(H)** Distribution of candidate target genes in AUROC–RMSE space.

We next evaluated dependency predictability across the analyzable pan-DepMap candidate set and summarized gene-level performance using AUROC and *R* ^2^ (Figure 5). Top genes by AUROC included ATP6V1B2, MYC, TUBB, PSMB6 and FERMT2 (Figure 5A), while top genes by *R* ^2^ included FERMT2, ADSL, CRKL, CHMP4B and CDAN1 (Figure 5B). AUROC–R^2^ scatterplots revealed a narrow high-performance region, and thresholded views indicated that only a small set of genes simultaneously achieves strong separability and predictability (Figures 5C–E).

To integrate these aspects, we defined DepScore = AUROC × max (R^2^, 0) and derived a compact candidate target set dominated by FERMT2, CRKL, MYC, CHMP4B, together with ADSL, CDAN1, CCND1, PSMB5/6, COPG1, PPP1R12A, YRDC and TUBB (Figure 5F). These genes also showed favorable multi-metric profiles (AUROC/R^2^/RMSE heatmap; Figure 5G) and clustered in the high-AUROC/low-RMSE regime (Figure 5H), motivating downstream biological assessment.

To address concerns about arbitrariness of threshold choice and potential random gene-set artifacts, we performed sensitivity analyses across AUROC and R^2^ thresholds, compared alternative composite ranking rules, and benchmarked against matched-size random gene sets (Supplementary Figure S1). The high-signal set showed stable overlap across threshold combinations, and recurrently prioritized genes such as FERMT2, CRKL, MYC, CHMP4B, and CCND1 remained prominent across several ranking rules. These analyses support the robustness of the candidate-set definition while also identifying DepScore as a pragmatic rather than mathematically optimal scoring rule.

To test whether model ranking was robust to partitioning strategy, we re-evaluated all benchmarked models using five-fold cross-validation on the high-signal gene benchmark set. The ranking was highly consistent with the original held-out benchmark. ExtraTrees achieved the best overall performance (mean AUROC 0.876 ± 0.012, mean R^2^ 0.236 ± 0.024, mean RMSE 0.240 ± 0.006), followed by SVR_RBF (0.862 ± 0.010, 0.207 ± 0.020, 0.247 ± 0.006) and XGBoost (0.847 ± 0.010, 0.169 ± 0.020, 0.259 ± 0.006). Linear baselines remained weaker, with Ridge showing the lowest mean R^2^ (−0.084 ± 0.015) and the highest mean RMSE (0.310 ± 0.004). The fold-level ExtraTrees-versus-Ridge difference in mean R^2^ is therefore approximately 0.32 in Supplementary Table S4, whereas the held-out paired ΔR^2^ summaries in Figures 3, 4 use gene-wise distributions and should not be conflated with this fold-level aggregate. These fold-wise results support the robustness of the benchmark ranking while clarifying the averaging unit used for the reported performance values.

### 3.4 TCGA multi-omics analyses support candidate target set coherence and set-level clinical association

**FIGURE 6:**
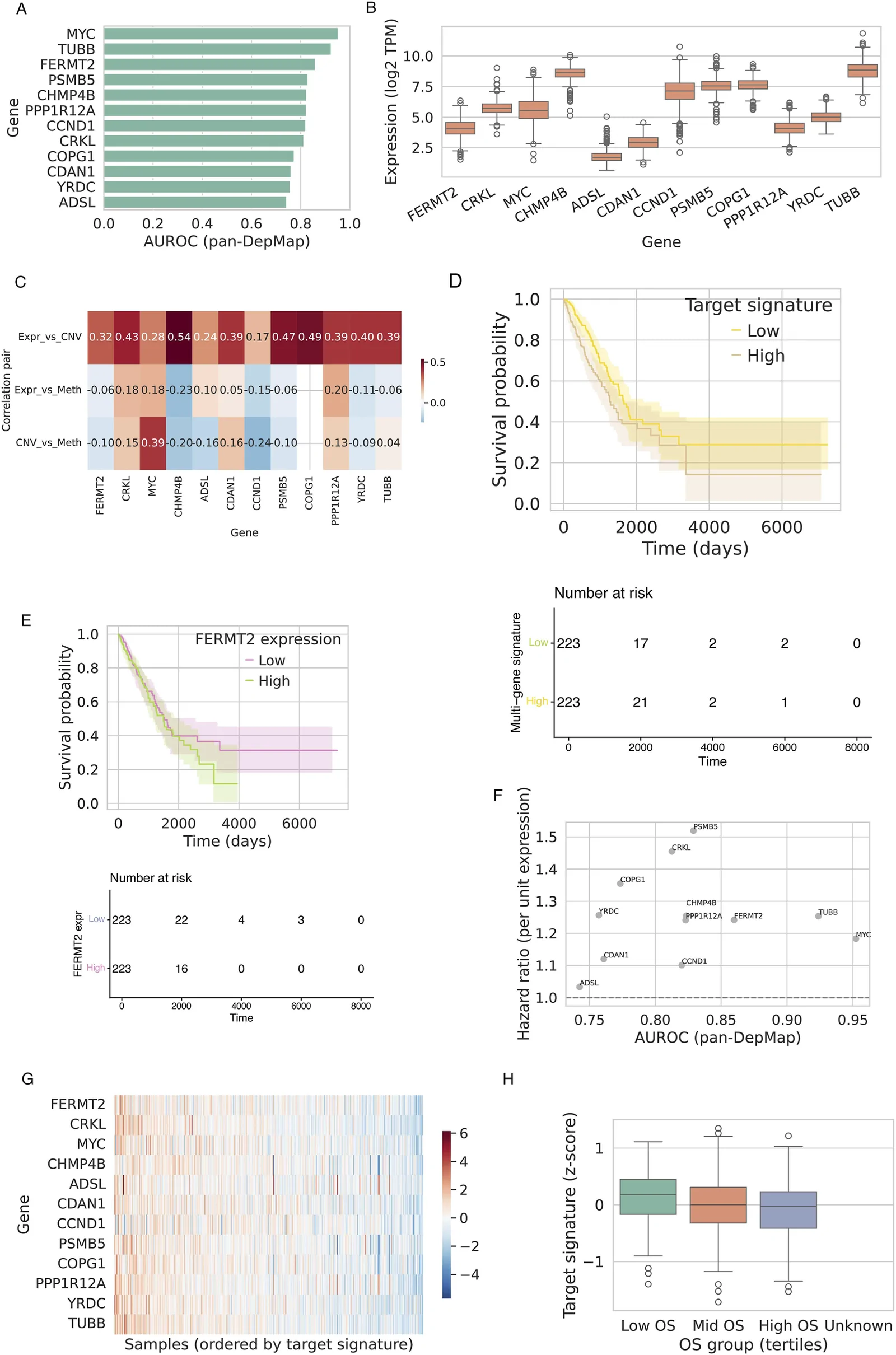
Multi-omics coherence and prognostic relevance of the DepPrior-prioritized candidate target set in TCGA-LUAD. **(A)** AUROC values of DepPrior-prioritized candidate target genes across pan-DepMap, highlighting their dependency predictability. **(B)** Expression levels (log2 TPM) of candidate target genes across TCGA-LUAD tumors. **(C)** Pairwise correlations between gene expression, copy-number variation and DNA methylation for candidate target genes. **(D)** Kaplan–Meier overall survival stratified by high versus low DepSignature in TCGA-LUAD. **(E)** Kaplan–Meier overall survival stratified by high versus low FERMT2 expression. **(F)** Relationship between dependency predictability (AUROC) and hazard ratios of candidate target genes. **(G)** Heatmap showing expression of candidate target genes across samples ordered by the DepSignature. **(H)** Distribution of the DepSignature across overall survival tertiles.

The prioritized genes retained high AUROC values in the pan-DepMap analysis (Figure 6A). We next tested whether the dependency-derived candidate target set forms a coherent, multi-omic module in primary LUAD tumors. In TCGA-LUAD, candidate target genes displayed coordinated expression structure and cross-omic coupling between expression, CNV and methylation (Figures 6B,C). Importantly, a multi-gene DepSignature showed stronger survival stratification than representative single-gene analyses (Figures 6D,E), most likely reflecting signal aggregation across a shared tumor-state axis. Several candidate target genes also showed a structured relationship between dependency predictability (AUROC) and hazard ratio estimates in TCGA (Figure 6F), consistent with a clinically associated tumor axis but not sufficient to establish causal dependency in patient tumors. The ordered expression heatmap and OS-tertile distributions further illustrated sample-level signature structure (Figures 6G,H).

**FIGURE 7:**
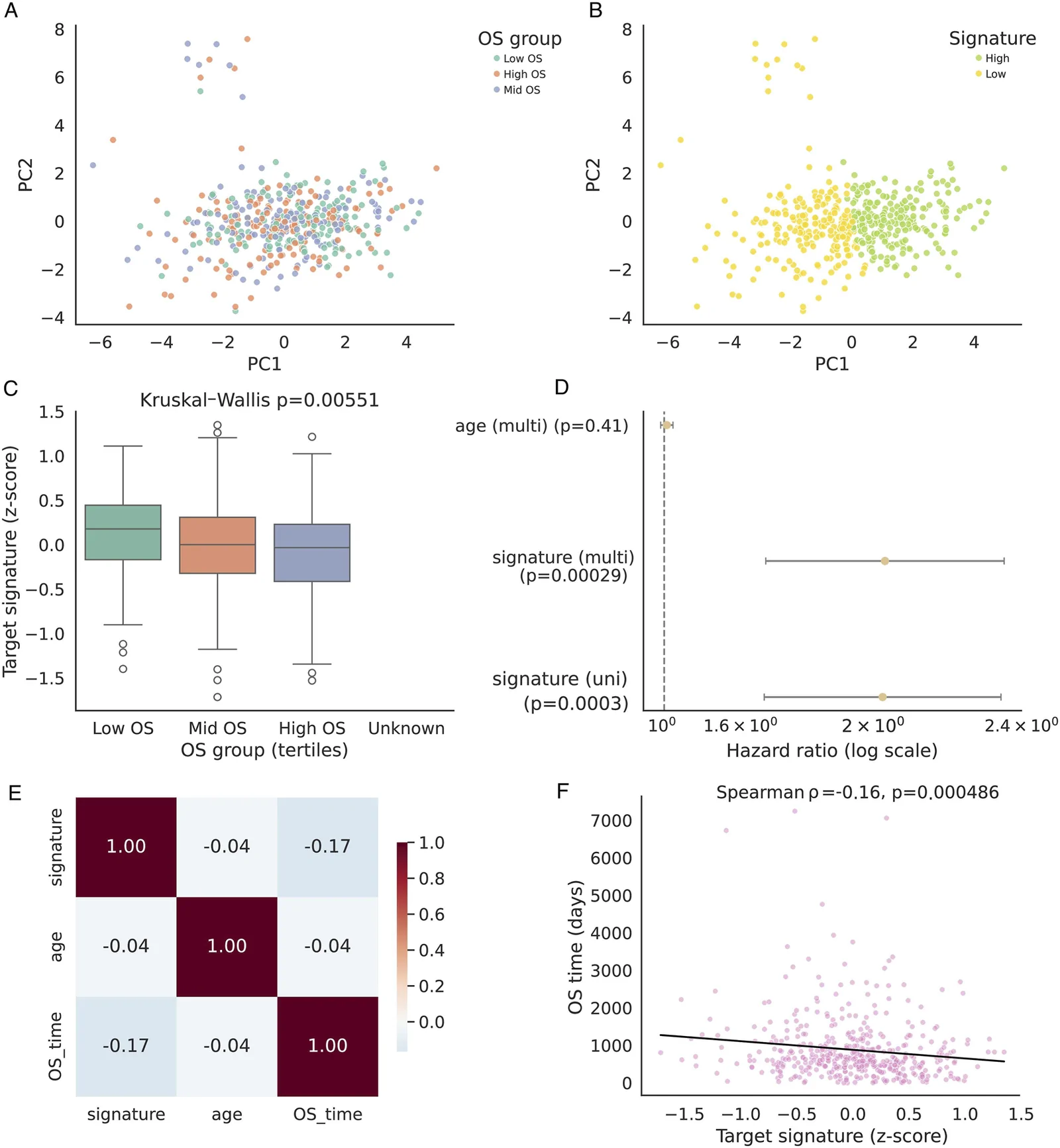
DepSignature captures a prognostic tumor axis in TCGA-LUAD. **(A)** Principal component analysis (PCA) of candidate target set gene expression. **(B)** PCA colored by DepSignature stratification. **(C)** Distribution of DepSignature across overall survival tertiles. **(D)** Cox proportional hazards analysis treating the signature as a continuous variable. **(E)** Summary of correlation analyses between the signature and clinical endpoints. **(F)** Correlation between DepSignature and overall survival time.

We further characterized the signature within TCGA. PCA of target genes revealed structured embeddings consistent with OS grouping and signature stratification (Figures 7A,B). The signature differed across OS tertiles (Kruskal-Wallis p = 0.00551) (Figure 7C). In Cox proportional hazards models treating the signature as a continuous variable, the signature was significant in both univariable (p = 0.0003) and multivariable analyses adjusting for age (signature multivariable p = 0.00029, age multivariable p = 0.41) (Figure 7D). Proportional-hazards diagnostics did not identify a material violation for the main signature model. Correlations among the signature, age, and OS time are summarized in Figure 7E. The signature also showed a modest negative association with survival time (Spearman rho = −0.16, p = 0.000486) (Figure 7F), indicating that the survival effect should be interpreted as exploratory and moderate in magnitude.

### 3.5 Biological interpretation: a proliferation-linked tumor-intrinsic candidate target set with staging associations

To interpret the candidate target set biologically, we examined canonical endpoints in TCGA-LUAD. The signature correlated strongly with proliferation markers, including MKI67 (Spearman ρ = 0.58, p = 1.66 × 10^−42^) and TOP2A (ρ = 0.60, p = 2.2 × 10^−45^) (Supplementary Figures S2A,B), and a composite proliferation score was significantly higher in signature-high tumors (Mann-Whitney p = 3.83 × 10^−31^; Supplementary Figure S2C). The signature also varied across AJCC N stage (Kruskal-Wallis p = 0.00657) and T stage (p = 0.00609) (Supplementary Figures S2D,E). In contrast, correlation with a simplified immune score was weak (Spearman ρ = 0.03, p = 0.59; Supplementary Figure S2F). Together, these results support a predominantly proliferation/cell-cycle and proteostasis-linked tumor-intrinsic axis, rather than an immune-dominant axis.

### 3.6 Genomic context: signature is elevated in TP53-mutant tumors and associates with selected driver alterations

We next examined whether the signature aligned with LUAD driver contexts. The signature did not differ significantly by EGFR or KRAS mutation status (EGFR: MW p = 0.22; KRAS: MW p = 0.97; Supplementary Figures S3A,B), but was significantly elevated in TP53-mutant tumors (MW p = 1.5 × 10^−10^; Supplementary Figure S3C). STK11 (MW p = 0.046) and ROS1 (MW p = 0.017) also showed significant associations (Supplementary Figures S3D,H), while KEAP1, BRAF, ALK and MET were not significant in the current cohort (Supplementary Figure S3E–G,I). These results support a genomic context consistent with a stress/proliferation-linked axis and provide an orthogonal line of evidence for biological plausibility.

### 3.7 DepMap cross-lineage analyses indicate lung-biased dependency patterns

**FIGURE 8:**
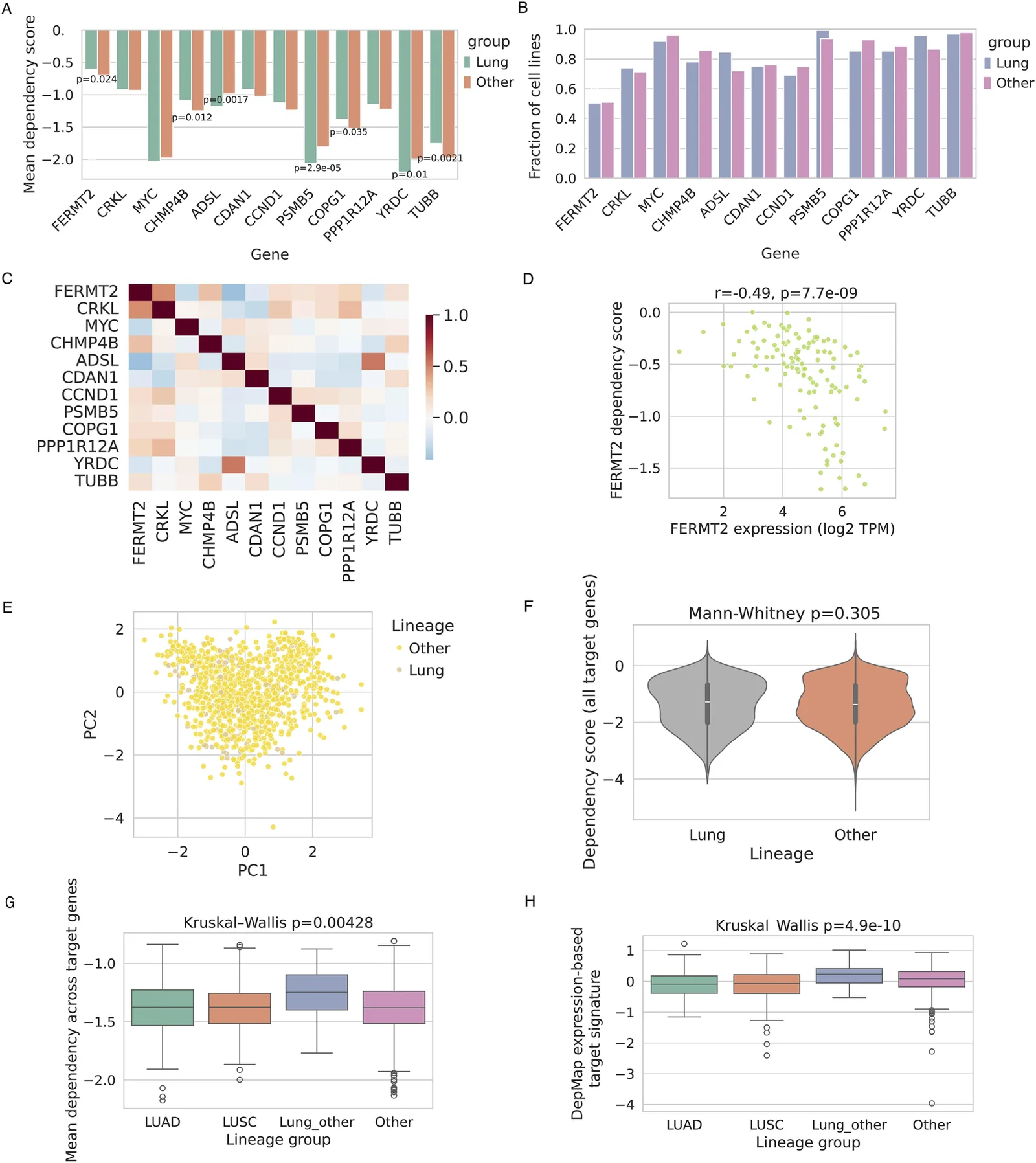
Cross-lineage dependency patterns of candidate target genes in DepMap. **(A)** Mean dependency scores of candidate target genes across lung and non-lung lineages. **(B)** Fraction of strongly dependent cell lines by lineage. **(C)** Co-dependency network structure of candidate target genes. **(D)** Relationship between expression and dependency for FERMT2 in lung cancer cell lines. **(E)** Principal component analysis of aggregated dependency profiles, colored by lung versus other lineage. **(F)** Comparison of dependency distributions between lung and other lineages. **(G)** Lineage-stratified comparison across LUAD, LUSC, lung-other and non-lung cell lines. **(H)** Expression-based DepSignature across lineage groups.

To assess lineage specificity, we compared dependencies across lung and non-lung lineages in DepMap. Candidate target genes displayed differences in mean dependency and strong-dependency fraction between lung and other lineages (Figures 8A,B), and co-dependency analysis revealed a coherent module structure (Figure 8C). For a representative gene (FERMT2), expression inversely correlated with dependency in lung cancer cell lines (r = −0.49, p = 7.7 × 10^−9^) (Figure 8D). PCA of aggregate dependency profiles did not show clear separation between lung and other lineages (Figure 8E). Although the aggregated dependency distribution over all candidate target genes showed only modest separation between Lung and other lineages (Mann–Whitney p = 0.305) (Figure 8F), lineage-stratified comparisons supported systematic differences across LUAD/LUSC/lung-other/other groups (Kruskal–Wallis p = 0.00428) (Figure 8G). An expression-based signature also differed strongly by lineage group (Kruskal–Wallis p = 4.9 × 10^−10^) (Figure 8H), reinforcing lung-linked candidate target set structure.

### 3.8 Functional enrichment supports cell-cycle, proteostasis and RTK–RAS signaling themes

**FIGURE 9:**
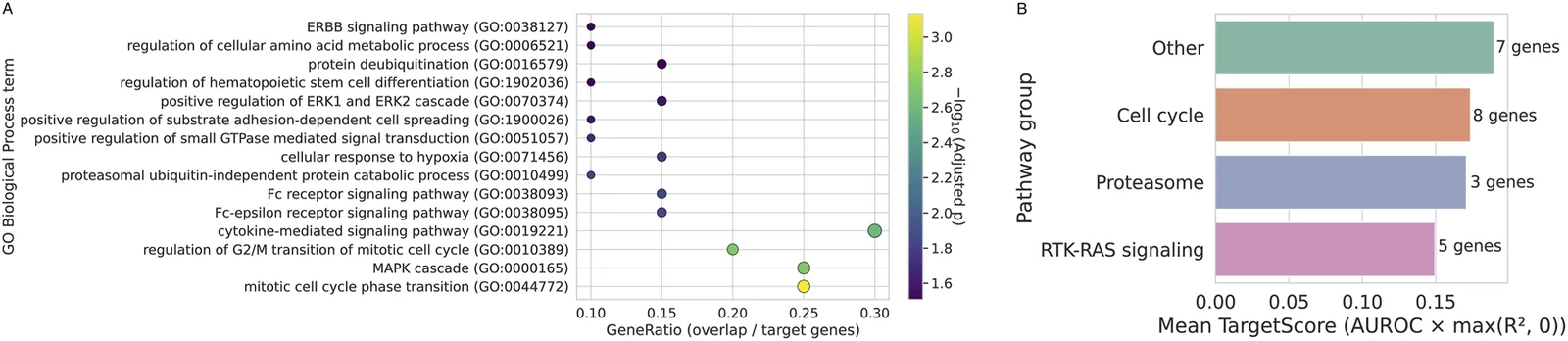
Functional enrichment of the DepPrior-prioritized candidate target set highlights proliferation and proteostasis pathways. **(A)** Gene Ontology biological process enrichment for candidate target genes. **(B)** Pathway-level grouping emphasizing cell cycle, proteostasis and RTK–RAS signaling modules.

Functional enrichment analyses of candidate target genes highlighted pathways consistent with proliferative state and proteostasis/stress responses. GO biological processes included terms related to cell-cycle transitions and signaling cascades, and pathway grouping emphasized cell cycle, proteasome/proteostasis, and RTK-RAS signaling modules (Figures 9A,B). These functional themes align with the candidate target set’s dependency predictability and its proliferation-linked behavior in tumors and cell lines.

### 3.9 External reproducibility assessment across GEO and CPTAC

**FIGURE 10:**
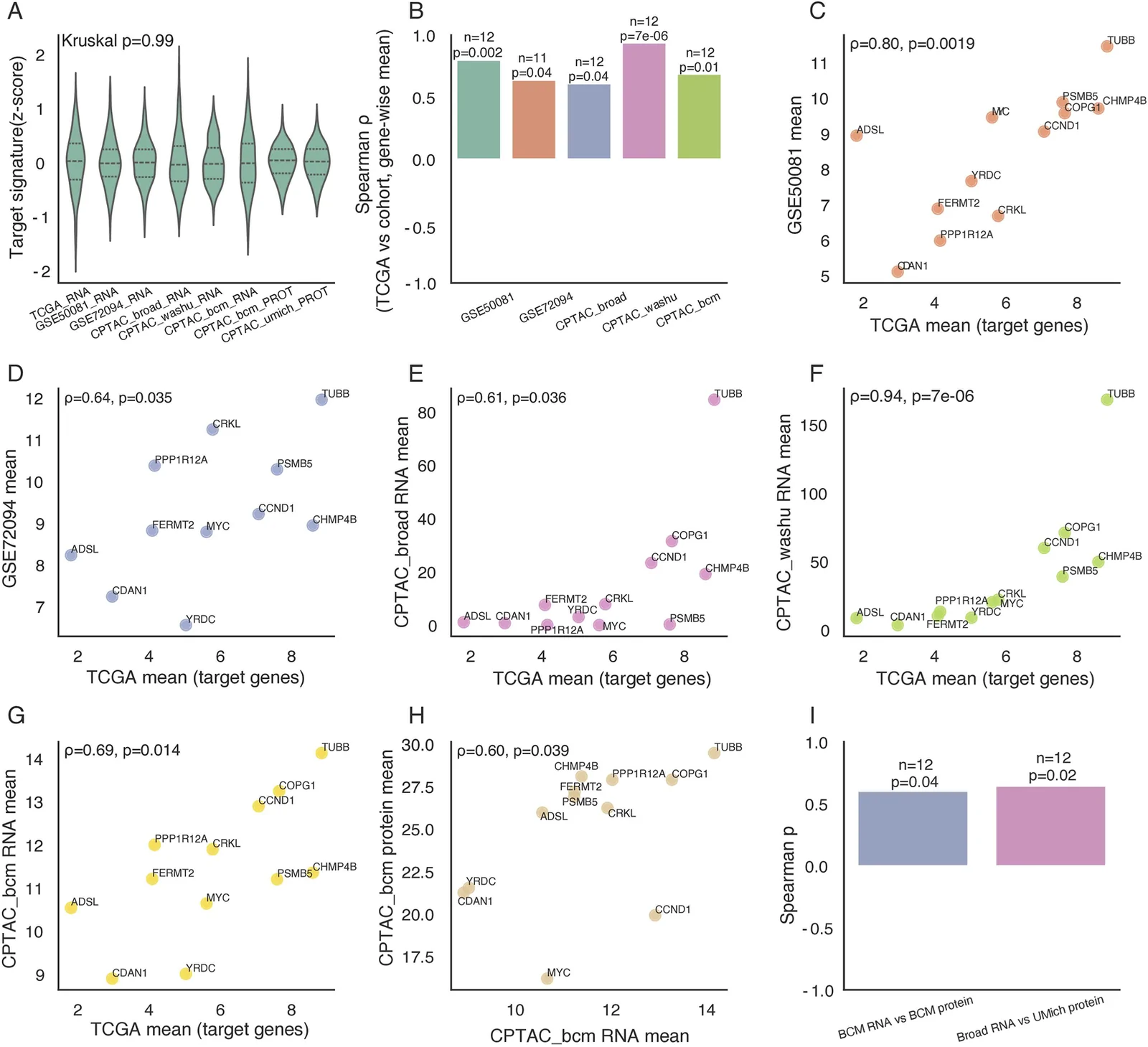
Cross-cohort reproducibility assessment of the candidate target set across TCGA, GEO, and CPTAC datasets. **(A)** Distribution of DepSignature (Z-score) across TCGA, GEO, and CPTAC RNA/protein cohorts, with no significant global difference assessed by the Kruskal-Wallis test. **(B)** Spearman correlations of candidate target gene expression between TCGA and external cohorts (GSE50081, GSE72094, CPTAC-Broad RNA, CPTAC-WashU RNA, and CPTAC-BCM RNA), with sample sizes and p-values indicated above bars. **(C–G)** Gene-wise concordance between TCGA mean expression of candidate target genes and their corresponding mean expression in independent cohorts: **(C)** GSE50081, **(D)** GSE72094, **(E)** CPTAC-Broad RNA, **(F)** CPTAC-WashU RNA, and **(G)** CPTAC-BCM RNA. Each point represents one candidate target gene, labeled accordingly, with Spearman correlation coefficients and p-values shown. **(H)** Correlation between CPTAC-BCM RNA and CPTAC-BCM protein mean expression levels for candidate target genes, demonstrating RNA–protein consistency. **(I)** Summary of Spearman correlations comparing RNA–protein concordance within CPTAC-BCM and RNA concordance between CPTAC-Broad and CPTAC-WashU cohorts, with sample sizes and p-values indicated.

Given that survival associations can be cohort-dependent in external datasets, we prioritized external assessment based on cross-cohort and cross-omics reproducibility of the candidate gene set. This analysis was designed to test expression and protein concordance, not to prove that cell-line dependencies are functionally conserved in patient tumors. The DepSignature showed comparable distributions across TCGA, two GEO cohorts (GSE50081, GSE72094), three CPTAC transcriptomics sources (Broad, WashU, BCM), and two CPTAC proteomics sources (BCM, UMich) (Kruskal p = 0.99) (Figure 10A), indicating cross-cohort stability of the measured signature.

At the gene level, mean expression ranks of candidate target genes were strongly concordant between TCGA and external RNA datasets, including GSE50081 (rho = 0.80, p = 0.0019) and GSE72094 (rho = 0.64, p = 0.035), as well as CPTAC transcriptomics sources (Broad rho = 0.61, p = 0.036; WashU rho = 0.94, p = 7 × 10^-6^; BCM rho = 0.69, p = 0.014) (Figures 10B–G). Candidate target set coherence also extended to the proteomic layer: RNA-protein concordance was significant in CPTAC-BCM (rho = 0.60, p = 0.039) (Figure 10H), and concordance between Broad transcriptomics and UMich proteomics was also significant (p = 0.02) (Figure 10I). These results support reproducible expression/protein structure across datasets, but they should not be interpreted as direct evidence of functional dependency conservation.

### 3.10 Orthogonal molecular support for apoptosis-associated response to FERMT2/CRKL perturbation

**FIGURE 11:**
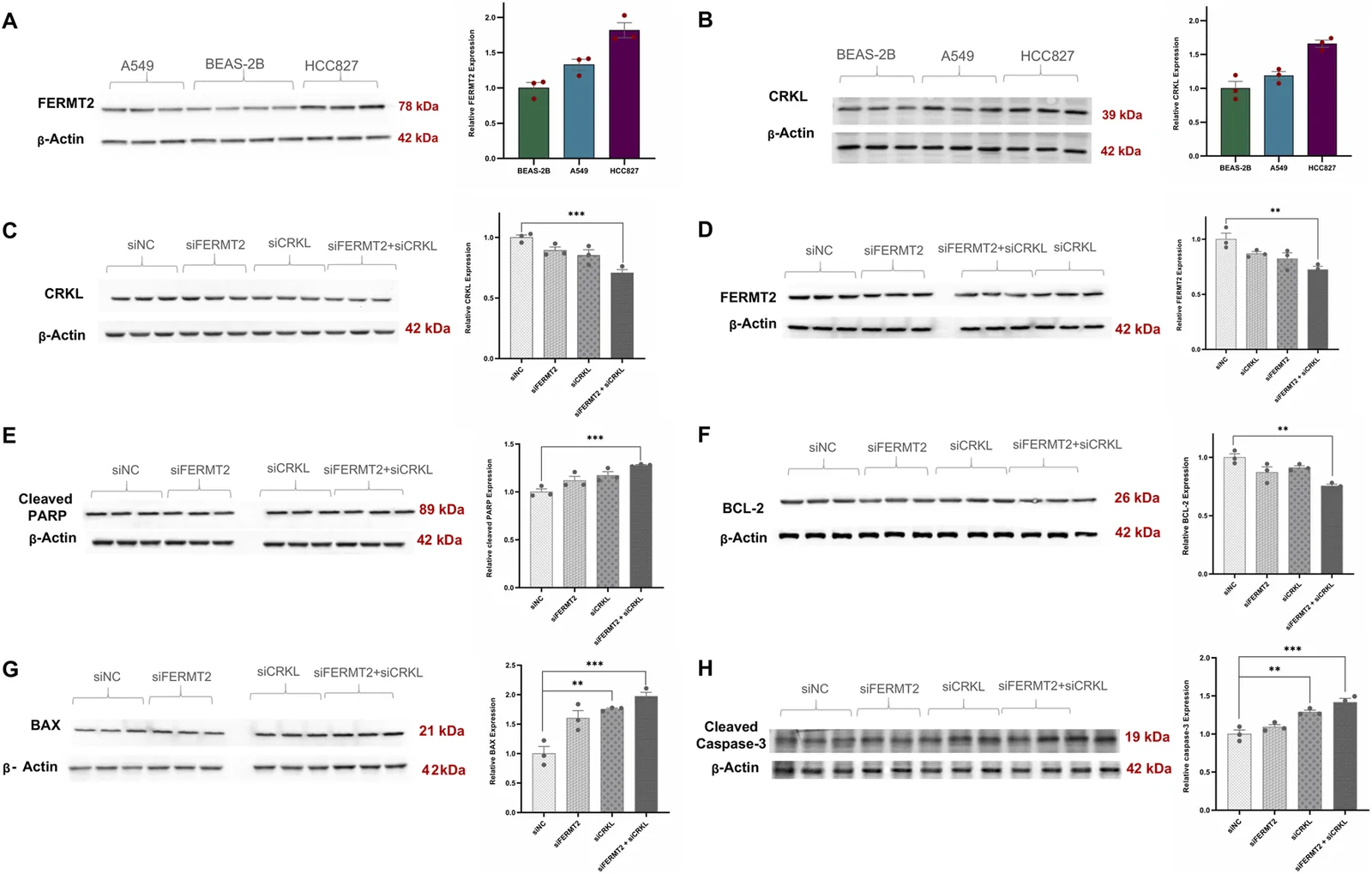
Western blot support for FERMT2/CRKL expression, siRNA-mediated knockdown, and apoptosis-associated protein changes in LUAD cells. **(A,B)** Representative western blots and densitometric quantification of basal FERMT2 and CRKL protein expression in BEAS-2B, A549, and HCC827 cells. Both FERMT2 and CRKL showed higher expression in LUAD cells, with the strongest expression observed in HCC827 cells. **(C,D)** Western blot analysis showed a modest reduction in FERMT2 and CRKL protein after siFERMT2, siCRKL, and combined siFERMT2 + siCRKL transfection in HCC827 cells compared with siNC controls, with the largest reduction after dual knockdown. **(E,F)** FERMT2 and/or CRKL perturbation was accompanied by increased apoptosis-related proteins cleaved PARP and cleaved caspase-3, with the most evident shift in the combined-knockdown group. **(G,H)** FERMT2/CRKL perturbation was accompanied by lower BCL-2 and higher BAX, particularly after combined FERMT2 and CRKL knockdown. These protein-marker changes should be interpreted as preliminary molecular support rather than definitive proof of on-target apoptotic pathway activation. β-actin was used as the loading control for all western blots. Quantitative data are presented as mean ± SD from three independent replicates; statistical significance is indicated in the figure as P < 0.05, P < 0.01, and P < 0.001. Consolidated uncropped/source-image plates are provided as Supplementary Source Data Figures WB-S1-WB-S8 and in the separate Western blot source-data supplementary file.

To test whether the computationally prioritized candidate target set was supported by orthogonal molecular evidence, we performed perturbation experiments targeting FERMT2 and CRKL in LUAD cell lines stratified by DepSignature level. Across the experimental panel, qRT-PCR indicated efficient mRNA-level knockdown, whereas immunoblotting showed a more modest reduction at the protein level in HCC827 cells (Figure 11). Baseline immunoblotting showed that both FERMT2 and CRKL were expressed most strongly in HCC827, more weakly in A549, and least in BEAS-2B (Figures 11A,B), supporting the use of HCC827 as a high-expression LUAD model for mechanistic interrogation.

In HCC827 cells, siFERMT2 produced a modest on-target reduction of FERMT2 protein, siCRKL produced a modest on-target reduction of CRKL protein, and combined knockdown produced the largest simultaneous reduction of both targets, although the overall magnitude of protein-level suppression was limited (Figures 11C,D). These data indicate a directionally on-target but partial effect at the protein level and provide a basis for interpreting the downstream apoptosis-marker changes with appropriate caution.

Target suppression in HCC827 was accompanied by a coordinated apoptosis-associated protein shift. Cleaved PARP and cleaved caspase-3 increased after single-gene knockdown and were highest in the combined-knockdown group, whereas BCL-2 decreased and BAX increased with the same overall pattern (Figures 11E–H). These data indicate that simultaneous FERMT2/CRKL suppression is accompanied by a stronger apoptosis-associated protein response than either single perturbation alone in this signature-high LUAD model, but they do not by themselves establish direct pathway activation or on-target causal dependency.

Together, the qRT-PCR and Western blot results provide orthogonal molecular support for representative DepPrior-prioritized genes and support the interpretation that combined FERMT2/CRKL inhibition in HCC827 cells is accompanied by a stronger apoptosis-associated protein response than either single perturbation alone. These data do not establish functional dependency for the full candidate set and do not replace rescue, proliferation, migration, clonogenic, or patient-derived model validation.

## 4 Discussion

In this study, we developed a predictability-guided framework for prioritizing a candidate LUAD therapeutic target set by integrating DepMap dependency profiles with tumor multi-omics and external transcriptomic/proteomic resources. The central idea was not that every measured dependency is equally informative, but that dependencies showing both separability and predictability may provide a more stable starting point for downstream target nomination. Within the currently analyzed candidate set, this strategy yielded a compact candidate target set centered on FERMT2, CRKL, MYC, CHMP4B, and related genes, with reproducible module-level support across TCGA, GEO, and CPTAC.

A strength of the study is its emphasis on reproducibility across data layers. Rather than relying exclusively on outcome association in a single cohort, we evaluated whether the prioritized genes form a coherent module across external transcriptomic and proteomic datasets. This is an appropriate assessment target for a computational prioritization framework. However, this strength should not be overstated: reproducible expression or protein-level structure across cohorts does not establish functional dependency conservation in patient tumors. The present work therefore supports candidate nomination, not definitive vulnerability validation.

An important biological insight is that the resulting signature is strongly aligned with proliferative tumor state. The association with MKI67, TOP2A, and stage-related variables suggests that the candidate target set is embedded in a broader proliferation/cell-cycle and proteostasis-related axis. This does not invalidate the candidate target set, because many therapeutically relevant vulnerabilities are coupled to proliferation-associated processes. At the same time, it limits claims of novelty. Our data are more consistent with a structured candidate dependency-derived target set associated with proliferative state than with the discovery of a wholly distinct and previously unrecognized LUAD vulnerability class.

The stronger survival stratification of the multi-gene signature is most plausibly explained by signal aggregation and noise reduction across correlated candidate target genes rather than by experimentally demonstrated functional synergy. In other words, combining multiple candidate target genes likely stabilizes the readout of a shared proliferative/dependency-associated tumor state and reduces the sensitivity of the analysis to noise in any single gene. Formal synergy testing will require perturbation-based combinatorial experiments and should not be inferred from retrospective survival association alone.

Notably, the added immunoblotting data provide protein-level support for the biological relevance of FERMT2 and CRKL in a representative signature-high LUAD model. In HCC827 cells, combined suppression produced stronger increases in cleaved PARP and cleaved caspase-3 together with lower BCL-2 and higher BAX than either single perturbation alone, indicating that the enhanced dual-perturbation condition was accompanied by an apoptosis-associated protein shift. At the same time, these data should not be overextended: they provide model-restricted molecular support for selected targets, not formal evidence of candidate-set-wide synergy, universal dependency conservation, or on-target specificity.

The translational tractability of the prioritized genes is also heterogeneous. Some components map to more actionable biological axes than others. For example, CCND1 points toward the CDK4/6 regulatory axis, which has been widely investigated therapeutically in solid tumors, including non-small cell lung cancer settings (Redman et al., 2020). Likewise, PSMB5 and PSMB6 suggest possible proteostasis-related vulnerabilities, although proteasome inhibition is not an established standard therapeutic strategy in LUAD. MYC remains a biologically central but pharmacologically difficult target, more plausibly approached through indirect or synthetic-lethal strategies than by straightforward direct inhibition (Garralda et al., 2024; Fukazawa et al., 2010). CRKL has context-specific relevance in lung cancer signaling biology, including in subsets of oncogene-driven disease, but is not yet a mature clinical target (Cheung et al., 2011). FERMT2 and several additional candidates in the candidate set are best regarded as mechanistically interesting and hypothesis-generating rather than immediately druggable targets.

This study has several limitations. First, the central predictive models were learned in cell lines and then interpreted in the context of primary tumors (Ben-David et al., 2018; Gillet et al., 2011). This transfer step is biologically non-trivial and likely incomplete because cell lines do not capture tumor microenvironmental effects, stromal interactions, immune pressure, drug exposure history, or patient-level clonal architecture. Second, the current implementation reflects the analyzable candidate pool available in the provided workflow; thus, claims should remain proportional to the scope of that scan unless a true genome-scale rerun is performed. Third, model improvements over linear baselines, while consistent, were moderate, and their biological importance should not be overstated. Fourth, the DepScore metric is heuristic and was not designed as a formally optimal statistical estimator. Fifth, orthogonal experiments were restricted to FERMT2 and CRKL in selected cell-line systems. Protein-level knockdown of FERMT2 and CRKL in HCC827 cells was modest, and no rescue experiments using re-expression or siRNA-resistant constructs were performed. Consequently, although the apoptosis-associated protein changes were directionally consistent and strongest after combined suppression, we cannot formally exclude that part of the observed response reflects off-target or transfection-associated stress rather than specific on-target dependency. These experiments should therefore be regarded as preliminary, model-restricted molecular support for selected representative genes. Establishing on-target specificity and functional dependency will require rescue experiments together with proliferation, clonogenic, migration, patient-derived model, and therapeutic-tractability assays before any definitive functional dependency claim is made.

Future work should address these limitations in four directions. First, a broader and more rigorously tuned predictive modeling framework should compare alternative composite scoring schemes and test whether the current product-based formulation is optimal. Second, orthogonal validation should be expanded across a larger LUAD cell-line panel and patient-derived systems, with rescue experiments used to distinguish on-target effects from broader perturbation-associated stress responses. Third, the biological interpretation of the candidate target set would benefit from mechanistic dissection of whether proliferation-linked association reflects direct dependency biology or broader tumor-state coupling. Fourth, subsequent public releases should preserve the reproducibility package through versioned archival snapshots, automated run checks on representative inputs, and, where feasible, an interactive Shiny or comparable web interface to improve accessibility for non-expert users.

## 5 Conclusion

DepPrior provides a predictability-guided computational framework for prioritizing candidate LUAD therapeutic targets by integrating DepMap dependency data with tumor multi-omics, cross-cohort reproducibility assessment, and initial perturbation-based experiments. The prioritized candidate target set shows coherent expression structure in TCGA-LUAD, reproducible module-level support across GEO and CPTAC resources, and preliminary protein-level support for representative genes in LUAD cells. These findings support its value as a hypothesis-generating candidate set for downstream experimental interrogation. Definitive functional, druggability, and therapeutic claims will require broader perturbation-based validation, rescue experiments, and patient-derived model testing.

## Data Availability

The datasets analyzed in this study are publicly available from TCGA/GDC (TCGA-LUAD; https://portal.gdc.cancer.gov/projects/TCGA-LUAD), DepMap (https://depmap.org/portal/download/all/), GEO (GSE50081: https://www.ncbi.nlm.nih.gov/geo/query/acc.cgi?acc=GSE50081; GSE72094: https://www.ncbi.nlm.nih.gov/geo/query/acc.cgi?acc=GSE72094), and CPTAC/PDC (https://proteomic.datacommons.cancer.gov/pdc/). Code, analysis materials, and the complete qRT-PCR source-data package (qPCR_raw_data.zip) are available at https://github.com/jizhang0114/DepPrior1. Additional source data, including uncropped western blot images, are provided in the Supplementary Material.
