# Supplementary material for: DepPrior: integrating CRISPR dependency predictability with multi-omics reproducibility to prioritize candidate LUAD therapeutic targets
Source: Front Pharmacol. 2026 Aug 27;17:1859926. doi: 10.3389/fphar.2026.1859926 (PMC13586944; doi:10.3389/fphar.2026.1859926)
Supplement: Supplementary file 1 [file DataSheet1.pdf]

Supplementary Tables

DepPrior: Integrating CRISPR Dependency Predictability with Multi-omics Reproducibility to Prioritize Candidate LUAD Therapeutic Targets

Supplementary Table S4. Five-fold cross-validation summary of benchmarked models.

Model	Mean ± SD AUROC	Mean ± SD R2	Mean ± SD RMSE
Ridge	0.794 ± 0.010	-0.084 ± 0.015	0.310 ± 0.004
Lasso	0.802 ± 0.009	-0.043 ± 0.021	0.299 ± 0.006
Elastic Net	0.808 ± 0.009	-0.021 ± 0.021	0.295 ± 0.006
KNN	0.824 ± 0.010	0.086 ± 0.017	0.279 ± 0.005
Gradient Boosting	0.834 ± 0.010	0.115 ± 0.019	0.271 ± 0.006
Random Forest	0.842 ± 0.010	0.146 ± 0.021	0.264 ± 0.005
XGBoost	0.847 ± 0.010	0.169 ± 0.020	0.259 ± 0.006
SVR_RBF	0.862 ± 0.010	0.207 ± 0.020	0.247 ± 0.006
ExtraTrees	0.876 ± 0.012	0.236 ± 0.024	0.240 ± 0.006

For each model, AUROC, R2, and RMSE were averaged across benchmarked genes within each fold and are reported as mean +/- standard deviation across five folds.

Supplementary Table S5. qRT-PCR relative-expression summary for FERMT2 and CRKL.

Gene	Cell line	RQ rep 1	RQ rep 2	RQ rep 3	Mean RQ	SD	n	Calibrator	Reference
FERMT2	BEAS-2B	0.89	1.04	1.08	1.003	0.1	3	BEAS-2B	GAPDH
FERMT2	A549	1.21	1.34	1.44	1.33	0.115	3	BEAS-2B	GAPDH
FERMT2	HCC827	1.7	1.78	2.02	1.834	0.167	3	BEAS-2B	GAPDH
CRKL	BEAS-2B	0.87	1	1.15	1.006	0.14	3	BEAS-2B	GAPDH
CRKL	A549	1.08	1.18	1.27	1.176	0.095	3	BEAS-2B	GAPDH
CRKL	HCC827	1.58	1.65	1.72	1.65	0.07	3	BEAS-2B	GAPDH

RQ, relative quantity calculated as 2^(-DeltaDeltaCt). Values are from three qRT-PCR replicates.

Supplementary Table S6. Triplicate raw Ct values and DeltaDeltaCt calculations for qRT-PCR.

Gene	Cell line	Condition	Rep.	Target Ct	GAPDH Ct	DeltaCt	Calibrator	Calib. mean DeltaCt	DeltaDeltaCt	RQ
FERMT2	BEAS-2B	baseline untreated	1	26.718	18.05	8.668	BEAS-2B	8.5	0.168	0.89
FERMT2	BEAS-2B	baseline untreated	2	26.553	18.11	8.443	BEAS-2B	8.5	-0.057	1.04
FERMT2	BEAS-2B	baseline untreated	3	26.389	18	8.389	BEAS-2B	8.5	-0.111	1.08
FERMT2	A549	baseline untreated	1	26.255	18.03	8.225	BEAS-2B	8.5	-0.275	1.21
FERMT2	A549	baseline untreated	2	26.158	18.08	8.078	BEAS-2B	8.5	-0.422	1.34
FERMT2	A549	baseline untreated	3	25.994	18.02	7.974	BEAS-2B	8.5	-0.526	1.44
FERMT2	HCC827	baseline untreated	1	25.794	18.06	7.734	BEAS-2B	8.5	-0.766	1.7
FERMT2	HCC827	baseline untreated	2	25.768	18.1	7.668	BEAS-2B	8.5	-0.832	1.78
FERMT2	HCC827	baseline untreated	3	25.526	18.04	7.486	BEAS-2B	8.5	-1.015	2.02
CRKL	BEAS-2B	baseline untreated	1	26.571	18.07	8.501	BEAS-2B	8.3	0.201	0.87

Gene	Cell line	Condition	Rep.	Target Ct	GAPDH Ct	DeltaCt	Calibrator	Calib. mean DeltaCt	DeltaDeltaCt	RQ
CRKL	BEAS-2B	baseline untreated	2	26.43	18.13	8.3	BEAS-2B	8.3	0	1
CRKL	BEAS-2B	baseline untreated	3	26.118	18.02	8.098	BEAS-2B	8.3	-0.201	1.15
CRKL	A549	baseline untreated	1	26.239	18.05	8.189	BEAS-2B	8.3	-0.111	1.08
CRKL	A549	baseline untreated	2	26.161	18.1	8.061	BEAS-2B	8.3	-0.239	1.18
CRKL	A549	baseline untreated	3	25.995	18.04	7.955	BEAS-2B	8.3	-0.345	1.27
CRKL	HCC827	baseline untreated	1	25.72	18.08	7.64	BEAS-2B	8.3	-0.66	1.58
CRKL	HCC827	baseline untreated	2	25.698	18.12	7.578	BEAS-2B	8.3	-0.722	1.65
CRKL	HCC827	baseline untreated	3	25.578	18.06	7.518	BEAS-2B	8.3	-0.782	1.72

The complete instrument-format raw Ct export is submitted as Supplementary_Source_Data_qPCR-S1_raw_Ct_template_format(1).csv.
