# Supplementary material for: DepPrior: integrating CRISPR dependency predictability with multi-omics reproducibility to prioritize candidate LUAD therapeutic targets
Source: Front Pharmacol. 2026 Aug 27;17:1859926. doi: 10.3389/fphar.2026.1859926 (PMC13586944; doi:10.3389/fphar.2026.1859926)
Supplement: Supplementary file 2 [file Supplementaryfile1.pdf]

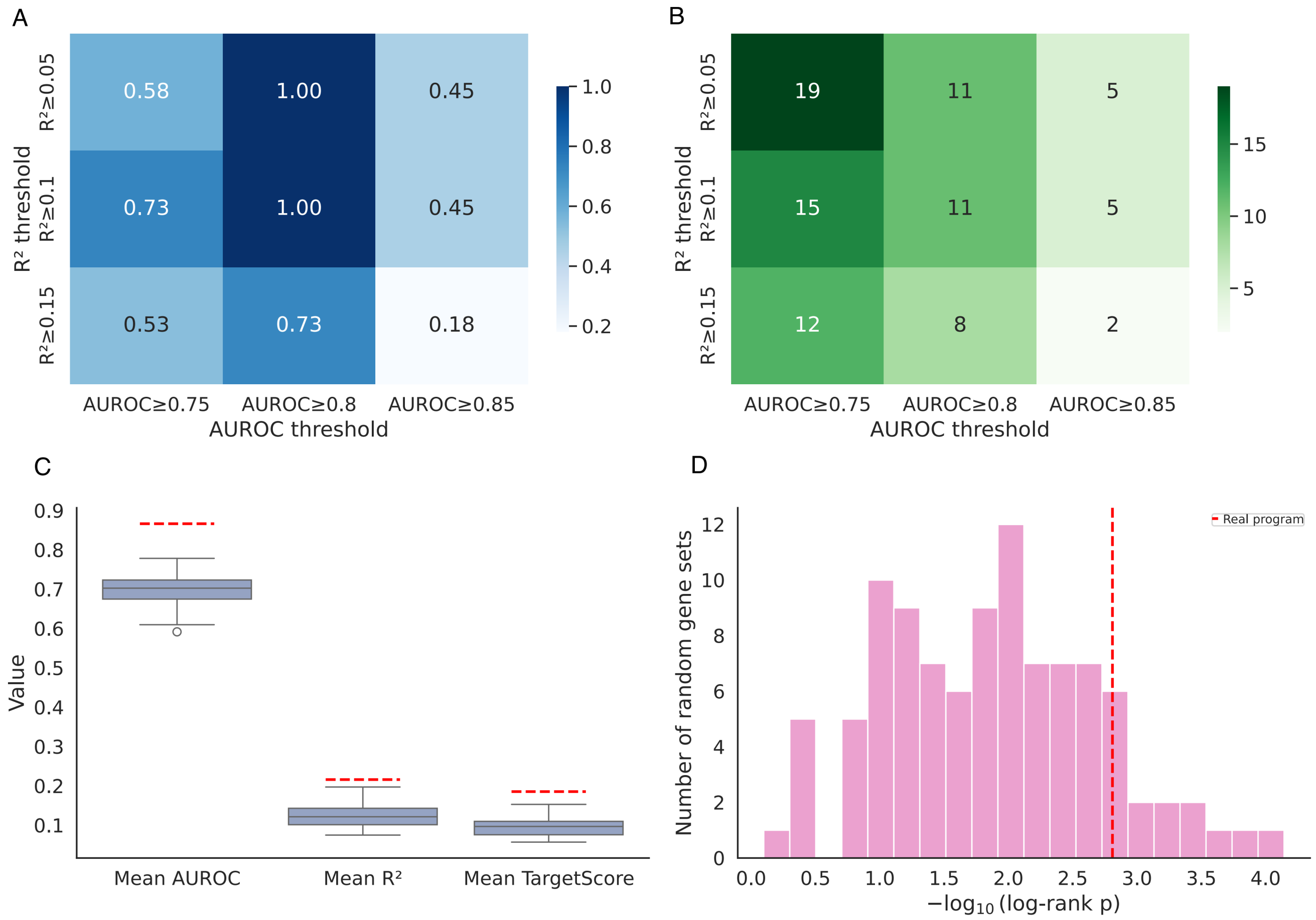

Figure S1. Performance landscape and significance assessment of DepSignature gene sets.

(A) Heatmap showing the proportion of DepSignature gene sets that satisfy combined AUROC and R² thresholds under different cutoff combinations. Columns indicate AUROC thresholds (≥ 0.75, ≥ 0.80, ≥ 0.85) and rows indicate R² thresholds (≥ 0.05, ≥ 0.10, ≥ 0.15). (B) Number of DepSignature gene sets passing the same AUROC and R² threshold combinations as in (A). (C) Distribution of mean AUROC, mean R², and mean DepScore values across all evaluated DepSignature gene sets. Red dashed lines indicate the observed values of the candidate target set. (D) Null distribution of -log10(log-rank p values) derived from random gene sets. The red dashed line denotes the -log10(log-rank p) of the observed DepSignature, demonstrating its significance compared with random background gene sets.

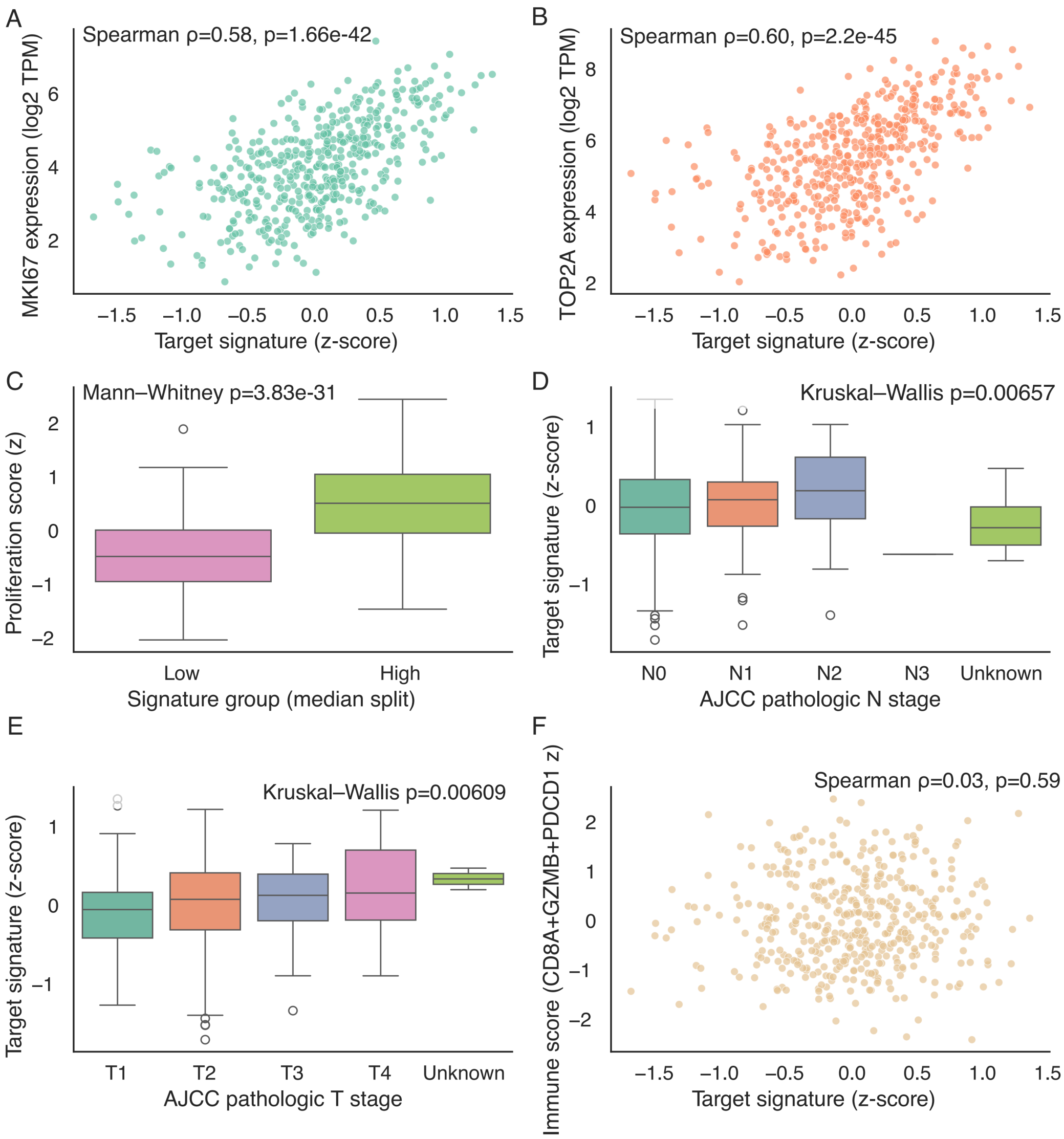

Figure S2. Clinical relevance of the DepSignature in TCGA-LUAD.

(A) Spearman correlation between MKI67 expression (log2 TPM) and DepSignature score (z-score). (B) Spearman correlation between TOP2A expression (log2 TPM) and DepSignature score (z-score). (C) Comparison of proliferation score (z-score) between low- and high-signature groups stratified by median split, with Mann-Whitney p value shown. (D) Distribution of DepSignature scores across AJCC pathologic N stages (N0-N3 and unknown), with Kruskal-Wallis p value indicated. (E) Distribution of DepSignature scores across AJCC pathologic T stages (T1-T4 and unknown), with Kruskal-Wallis p value indicated. (F) Spearman correlation between immune score (CD8A+GZMB+PDCD1 z-score) and DepSignature score (z-score).

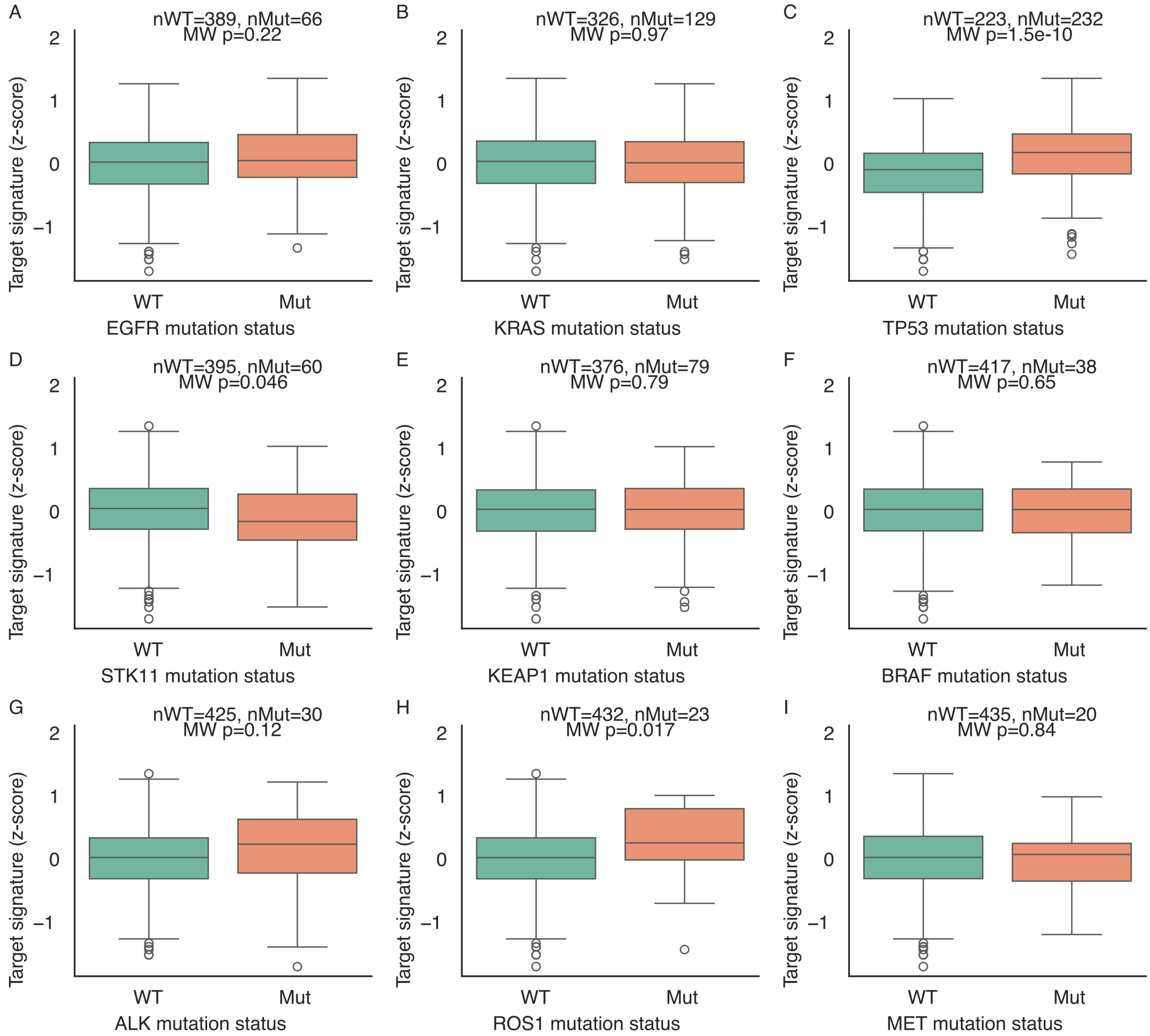

Figure S3. Association between DepSignature and somatic mutation landscape in TCGA-LUAD.

(A) DepSignature scores stratified by EGFR mutation status. (B) DepSignature scores stratified by KRAS mutation status. (C) DepSignature scores stratified by TP53 mutation status. (D) DepSignature scores stratified by STK11 mutation status. (E) DepSignature scores stratified by KEAP1 mutation status. (F) DepSignature scores stratified by BRAF mutation status. (G) DepSignature scores stratified by ALK mutation status. (H) DepSignature scores stratified by ROS1 mutation status. (I) DepSignature scores stratified by MET mutation status.

Supplementary Table S4. Five-fold cross-validation summary of benchmarked models.

Model	Mean ± SD AUROC	Mean ± SD R2	Mean ± SD RMSE
Ridge	0.794 ± 0.010	-0.084 ± 0.015	0.310 ± 0.004
Lasso	0.802 ± 0.009	-0.043 ± 0.021	0.299 ± 0.006
Elastic Net	0.808 ± 0.009	-0.021 ± 0.021	0.295 ± 0.006
KNN	0.824 ± 0.010	0.086 ± 0.017	0.279 ± 0.005
Gradient Boosting	0.834 ± 0.010	0.115 ± 0.019	0.271 ± 0.006
Random Forest	0.842 ± 0.010	0.146 ± 0.021	0.264 ± 0.005
XGBoost	0.847 ± 0.010	0.169 ± 0.020	0.259 ± 0.006
SVR_RBF	0.862 ± 0.010	0.207 ± 0.020	0.247 ± 0.006
ExtraTrees	0.876 ± 0.012	0.236 ± 0.024	0.240 ± 0.006

For each model, AUROC, R2, and RMSE were averaged across benchmarked genes within each fold and are reported as mean ± standard deviation across five folds.

Supplementary Table S5. qRT-PCR relative-expression summary for FERMT2 and CRKL.

Gene	Cell line	RQ rep 1	RQ rep 2	RQ rep 3	Mean RQ	SD	n	Calibrator	Reference
FERMT2	BEAS-2B	0.89	1.04	1.08	1.003	0.1	3	BEAS-2B	GAPDH
FERMT2	A549	1.21	1.34	1.44	1.33	0.115	3	BEAS-2B	GAPDH
FERMT2	HCC827	1.7	1.78	2.02	1.834	0.167	3	BEAS-2B	GAPDH
CRKL	BEAS-2B	0.87	1	1.15	1.006	0.14	3	BEAS-2B	GAPDH
CRKL	A549	1.08	1.18	1.27	1.176	0.095	3	BEAS-2B	GAPDH
CRKL	HCC827	1.58	1.65	1.72	1.65	0.07	3	BEAS-2B	GAPDH

RQ, relative quantity calculated as 2^(-DeltaDeltaCt). Values are from three qRT-PCR replicates.

Supplementary Table S6. Triplicate raw Ct values and DeltaDeltaCt calculations for qRT-PCR.

Gene	Cell line	Condition	Rep.	Target Ct	GAPDH Ct	DeltaCt	Calibrator	Calib. mean DeltaCt	DeltaDeltaCt	RQ
FERMT2	BEAS-2B	baseline untreated	1	26.718	18.05	8.668	BEAS-2B	8.5	0.168	0.89
FERMT2	BEAS-2B	baseline untreated	2	26.553	18.11	8.443	BEAS-2B	8.5	-0.057	1.04
FERMT2	BEAS-2B	baseline untreated	3	26.389	18	8.389	BEAS-2B	8.5	-0.111	1.08
FERMT2	A549	baseline untreated	1	26.255	18.03	8.225	BEAS-2B	8.5	-0.275	1.21
FERMT2	A549	baseline untreated	2	26.158	18.08	8.078	BEAS-2B	8.5	-0.422	1.34
FERMT2	A549	baseline untreated	3	25.994	18.02	7.974	BEAS-2B	8.5	-0.526	1.44
FERMT2	HCC827	baseline untreated	1	25.794	18.06	7.734	BEAS-2B	8.5	-0.766	1.7
FERMT2	HCC827	baseline untreated	2	25.768	18.1	7.668	BEAS-2B	8.5	-0.832	1.78
FERMT2	HCC827	baseline untreated	3	25.526	18.04	7.486	BEAS-2B	8.5	-1.015	2.02
CRKL	BEAS-2B	baseline untreated	1	26.571	18.07	8.501	BEAS-2B	8.3	0.201	0.87
CRKL	BEAS-2B	baseline untreated	2	26.43	18.13	8.3	BEAS-2B	8.3	0	1
CRKL	BEAS-2B	baseline untreated	3	26.118	18.02	8.098	BEAS-2B	8.3	-0.201	1.15
CRKL	A549	baseline untreated	1	26.239	18.05	8.189	BEAS-2B	8.3	-0.111	1.08
CRKL	A549	baseline untreated	2	26.161	18.1	8.061	BEAS-2B	8.3	-0.239	1.18
CRKL	A549	baseline untreated	3	25.995	18.04	7.955	BEAS-2B	8.3	-0.345	1.27
CRKL	HCC827	baseline untreated	1	25.72	18.08	7.64	BEAS-2B	8.3	-0.66	1.58
CRKL	HCC827	baseline untreated	2	25.698	18.12	7.578	BEAS-2B	8.3	-0.722	1.65
CRKL	HCC827	baseline untreated	3	25.578	18.06	7.518	BEAS-2B	8.3	-0.782	1.72

The complete instrument-format raw Ct export is submitted as Supplementary_Source_Data_qPCR-S1_raw_Ct_template_format(1).csv.
