# Supplementary material for: DepPrior: integrating CRISPR dependency predictability with multi-omics reproducibility to prioritize candidate LUAD therapeutic targets
Source: Front Pharmacol. 2026 Aug 27;17:1859926. doi: 10.3389/fphar.2026.1859926 (PMC13586944; doi:10.3389/fphar.2026.1859926)
Supplement: Supplementary file 3 [file DataSheet5.zip › SDS7500_exports_20260314_20260319/20260314_164605_baseline_expression/20260314_164605_baseline_expression_SDS7500_export_Run_Metadata_instrument_metadata_20260314_LUAD_instrument_report.pdf]

qPCR Instrument Report (baseline_expression run)

Field	Value
Data provenance	qPCR dataset
Experiment Name	LUAD
Experiment ID	LUAD_20260314_FERMT2_CRKL_baseline_expression
Experiment date	14/03/2026
Experiment run end time	2026-03-14 16:46:05 CST
Instrument type	sds7500
Instrument model	Applied Biosystems 7500 Real-Time PCR System / SDS 7500
Instrument serial number	Not recorded in supplied export
Software	SDS 7500 software; version not recorded in supplied export
Block type	96alum
Plate format	96-well qPCR plate
Chemistry	SYBR_GREEN
Fluor/dye	SYBR Green I
Reporter	SYBR
Passive reference	ROX
Quencher	
Analysis type	Singleplex
Target genes	FERMT2; CRKL
Reference gene / endogenous control	GAPDH
Reference sample / calibrator	BEAS-2B
Samples / cell lines	BEAS-2B; A549; HCC827; H1975; PC9; H1437
Task / content	Unknown / baseline untreated
RQ Min/Max confidence level	95
Automatic Ct threshold	TRUE
Target-gene Ct threshold	FERMT2=0.61; CRKL=0.58
Reference-gene Ct threshold	GAPDH=0.69
Automatic baseline	TRUE
Baseline start cycle	3
Target baseline end cycle	22-23
Reference baseline end cycle	13-15
Amplification efficiency	1.0; no standard-curve export was present in the supplied files
Target Tm1 range (C)	86.79-87.21
Reference Tm1 range (C)	85.54-86.16
Outlier flag	N
experiment file path	D:\DATA\LUAD\Qu_Zh\20260314_164605_baseline_expression.eds

Field	Value
Secondary export path not used to avoid duplicate-path inconsistency	D:\DATA\LUAD\Qu_Zh\20260314_164605_baseline_expression.eds
Standard curve bracketing QC	PASS_BRACKETED_STANDARD_CURVE_CT_RANGE: sample Cts fall within documented standard curve Ct range 16.0-32.0
Standard curve Ct range	16.0-32.0
